# *In vitro* and *in silico* modelling of ROS1‐positive non‐small cell lung cancer reveals fusion‐dependent tyrosine kinase inhibitor responses

**DOI:** 10.1002/1878-0261.70291

**Published:** 2026-06-23

**Authors:** Farhan Ul Haq, Marc Terrones, Felicia Rodrigues Fortes, Anne Schepers, Alessia Denis, Christophe Deben, Ken Op de Beeck, Guy Van Camp, Geert Vandeweyer

**Affiliations:** ^1^ Center for Oncological Research University of Antwerp, and Antwerp University Hospital Wilrijk Belgium; ^2^ Center of Medical Genetics University of Antwerp and Antwerp University Hospital Edegem Belgium

**Keywords:** CRISPR/Cas9, molecular dynamics simulations, protein modelling, ROS1+ NSCLC, TKI‐resistant patient‐derived cell lines, tyrosine kinase inhibitors

## Abstract

The emergence of drug resistance in ROS1‐rearranged non‐small cell lung cancer (NSCLC) represents a major therapeutic challenge. Although several resistance mutations have been described in patients, preclinical models derived from patient material remain limited, thereby hindering mechanistic insight into this malignancy. In this study, we investigated three clinically relevant ROS1 variants (G2032R, L2026M, and S1986Y) using CRISPR/Cas9‐edited patient‐derived cell lines and complemented these analyses with molecular docking and molecular dynamics simulations. The efficacy of tyrosine kinase inhibitors (TKIs) crizotinib, ceritinib, lorlatinib, entrectinib, and repotrectinib was systematically evaluated. Dose–response assays with CUTO‐28 (TPM3–ROS1) and CUTO‐37 (CD74–ROS1) lines reproduced clinical drug responses, revealing fusion‐dependent differences in resistance. The G2032R mutation led to reduced sensitivity to entrectinib and lorlatinib, with lower area‐over‐the‐curve values compared with ROS1 wild‐type (WT) cells. Similar reductions were observed for L2026M and S1986Y, while complete resistance was only seen in CUTO‐37 G2032R. Immunoblotting confirmed impaired inhibition of p‐ROS1 (Tyr2274) in resistant models. Structural modelling revealed alterations in kinase active site pocket volume, activation helix rotation, and activation loop dynamics in ROS1 mutants, providing a mechanistic basis for the observed drug responses. Molecular dynamics simulations validated the type I inhibitor binding mode across ROS1 WT and mutant complexes, while highlighting conformational effects extending beyond direct ligand interactions. Our findings underscore that although G2032R and L2026M mutations reside within the kinase active site, their impact extends far beyond steric hindrance, altering overall kinase domain dynamics. Collectively, these data establish a robust panel of patient‐derived ROS1 cell lines that recapitulate clinical resistance patterns and, together with complementary computational modeling, provide a valuable framework to dissect ROS1 tumor biology and support rational design of next‐generation inhibitors.

## Graphical abstract

Drug resistance limits treatment success in a subset of lung cancers driven by ROS1 gene alterations. Using patient‐derived cells and computer simulations, we studied three key mutations and how they affect five targeted drugs. The mutations reduced drug effectiveness in different ways by altering protein structure and behavior. These findings improve understanding of resistance and may guide development of more effective future therapies.

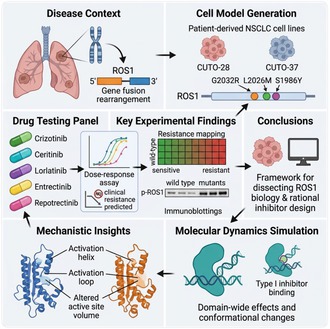

**Abbreviations**
NGR: Normalized Growth Rate
NSCLC: non‐small cell lung cancer
OS: overall survival
PDB: Protein Data Bank
PDCs: patient‐derived cell lines
PFS: progression‐free survival
ROS1: ROS proto‐oncogene 1, receptor tyrosine kinase
TKIs: tyrosine kinase inhibitors

## 1 Introduction

Targeted therapy has revolutionized cancer treatment in recent years, particularly for malignancies driven by aberrant kinase activity [1, 2]. Receptor tyrosine kinases (RTKs) play a critical role in cellular signaling, growth, differentiation, and other essential processes that maintain normal cellular function and survival [3]. Dysregulation or abnormal activity of these kinases can lead to cancer development, which can be effectively targeted with tyrosine kinase inhibitors (TKIs) [4]. The use of TKIs has shown significant success in treating cancers associated with Neurotrophic Tyrosine Receptor Kinase (NTRK), RET, Anaplastic Lymphoma Kinase (ALK), and c‐ros Oncogene 1 (ROS1) alterations [5, 6, 7].

ROS1 is an RTK, and genetic rearrangements in this gene lead to the production of chimeric ROS1 fusion proteins [8]. These fusion proteins act as biomarkers for ROS1+ non‐small cell lung cancer (NSCLC), a subtype that comprises approximately 1–2% of newly diagnosed NSCLC cases [9]. Patients with ROS1+ NSCLC typically exhibit a favorable response to TKI therapy, achieving longer progression‐free survival (PFS) and overall survival (OS) compared to those with other NSCLC subtypes [9, 10, 11, 12, 13].

However, despite the initial success of TKI therapy, most patients eventually relapse due to the emergence of resistance mutations, which render the inhibitors ineffective [14, 15, 16]. Aggravating these relapses are the rarity of ROS1+ NSCLC and its relatively recent discovery, which have limited the development and global approval of effective drugs [12, 17, 18]. Disparities in drug approval between different regions further exacerbate the challenge.

The scarcity of data on ROS1 fusions and their functional significance pose significant challenges for research. There is an urgent need for comprehensive experimental insights into both the structural and functional properties of native oncogenic ROS1‐fusion (termed wild‐type (WT)) and its therapy resistant (mutant) variants. Currently, no detailed information is available for any ROS1 mutations. Additionally, the limited availability of relevant cellular models further hampers efforts to understand the functional implications of ROS1 mutations and their role in disease progression and TKI resistance.

To overcome this limitation in preclinical models, genetic modification of those patient‐derived cell lines that are available can help in characterizing pathological mechanisms and drug resistance events. As a starting point, the CUTO collection of cell lines together with HCC78 constitute a representative panel of ROS1+ NSCLC lines driven by different rearrangements, thus reflecting the diversity of molecular subtypes [19]. Given that these lines do not harbor kinase domain mutations, they display a TKI‐sensitive phenotype. To assess the impact of some of the most common intrinsic resistance‐causing mutations in detail, CRISPR/Cas9‐mediated genome engineering can be applied to introduce these mutations.

In a previous study, we provided a proof of concept on using CRISPR/Cas9 on ROS1+ NSCLC cell models, namely the HCC78 cell model [20]. Moreover, we introduced a more accurate growth rate‐based metric (normalized growth rate, NOGR) to monitor the response toward compounds, discriminating between cytostatic and cytotoxic effects of TKIs. However, these results can be extrapolated only to the SLC34A2‐ROS1+ NSCLC patient cohort; consequently, we knocked in the ROS1 G2032R, L2026M, and S1986Y mutations in CUTO28 (TPM3‐ROS1) and CUTO37 (CD74‐ROS1) cell lines to extend our panel of TKI‐resistant patient‐derived cell lines and to profile their response against 5 clinically approved TKIs: crizotinib, entrectinib, lorlatinib, ceritinib, and repotrectinib.

Since the recognition of ROS1 fusions as biomarkers in NSCLC, extensive research has been devoted to unraveling the underlying biochemistry of this cancer. Additionally, considerable efforts have been invested in acquiring experimental structures for ROS1. Presently, only a handful of ROS1 structures have been deposited in the Protein Data Bank (PDB) [21] all representing the kinase's active state. From what has been published so far, mechanisms of known resistance mutations against Type I TKIs (G2032R [22], L2026M [23], and S1986Y [24]) can be mechanistically summarized as steric hindrance, restricted access to the enzyme's active site, alterations in electrostatic forces on the ATP‐binding site's outer surface, and modifications to the inhibitor binding pocket [25]. The experimental data in the form of complex structures of ROS1 mutant kinase domains bound to TKIs, which would provide a deeper understanding of the resistance profile associated with these mutations, are however lacking.

In our previous research, we focused on two mutation hotspots at positions 1982 and 1986, both of which lack experimental structural data, and their resistance mechanisms remain under investigation [26]. In these scenarios, lacking experimental structural information, homology modelling and molecular dynamics (MD) simulations have proven to be particularly valuable in elucidating the structural and dynamic characteristics of ROS1/TKI complexes. While numerous studies have identified factors providing some mechanistic insights, substantial work is still required in this area [16, 27, 28].

Our recent work centered on crizotinib in both a treatment naive (WT) ROS1 fusion and therapy resistant (mutant) ROS1 fusions, exploring ROS1 kinase domain dynamics. Furthermore, we expanded our inquiry to offer a comparative analysis of existing compounds used in treating ROS1+ NSCLC patients. This analysis aimed to discern commonalities and differences among these compounds, providing insights into resistance mechanisms and aiding in the development of improved compounds capable of circumventing mutation‐induced challenges.

In this study, we are leveraging existing information and expanding our computational investigation, complementing experimental findings. Hence, this study aimed to integrate cellular responses with structural data in a preclinically relevant experimental fashion, being a more refined approach that bridges the gap between the limited availability of patient‐derived models and the clinical setting. We propose that the ATP‐pocket mutations have a broader implication beyond simple steric hindrance and active site changes, involving a complex interplay that alters the dynamics of the G‐loop, αC‐helix, A‐loop, and crucially, the roles of HRD and DFG motifs in conferring resistance to type I TKIs.

In parallel, the study of these mutations within a cellular context contributes to the understanding beyond kinase domain dynamics, mediated by the role of the different ROS1 fusion gene partners, known to differentially modulate the ROS1‐mediated signaling.

## 2 Material and methods

### 2.1 Patient‐derived ROS1+ NSCLC cell lines

CUTO‐28 (driven by a TPM3‐ROS1 rearrangement) (RRID:CVCL_C8T2) and CUTO‐37 (CD74‐ROS1) (RRID:CVCL_C8T3) were kindly provided by Prof. Dr. Robert C. Doebele, as a result of the collaboration within the ‘The ROS1 cancer model project’ [29]. Cells were cultured in RPMI 1640 supplemented with 10% fetal bovine serum (v/v) and 1% L‐glutamine (v/v), in a humidified incubator at 37°C, 5% CO_2_. All lines used in this study were cultured under passage 30, authenticated via STR analysis within past 3 years and targeted sequencing of the *ROS1* rearrangement as well as routinely tested for Mycoplasma infection.

### 2.2 CRISPR/Cas9‐mediated genetic engineering

The protocol followed to generate the mutant lines has been previously reported by our group [20], except for the use of the EH‐100 nucleofection program combined with the SE solution (Lonza) to electroporate 1 × 10^6^ CUTO‐28 and CUTO‐37 cells per reaction.

### 2.3 Drug screening

The TKIs crizotinib (Selleck Chemicals, S1068), entrectinib (TargetMol Chemicals, T3670), lorlatinib (Merck, PZ0039), ceritinib (TargetMol Chemicals, T1791), and repotrectinib (Selleck Chemicals, S8583) were used for *in vitro* drug screening. Drug screening was performed at the DrugVision.AI automated screening facility at the University of Antwerp, Belgium. For two‐dimensional (2D) drug screening, 500 cells were plated in a 384‐well microplate (Corning, #3764) using an OT‐2 pipetting robot (Opentrons) and settled overnight. All drugs and fluorescent reagents were added to the plate using the Tecan D300e Digital Dispenser and dissolved in DMSO or 0.3% Tween‐20/H2O. Cytotox Green (60 nm/well, Sartorius, DMSO) was used as a fluorescent cell death marker and Staurosporine (2 μm, Tocris Bioscience, DMSO) as a positive control. For each drug, a 7‐point logarithmic titration was dispensed (1–5000 nm), and DMSO concentrations were normalized to the same level in each well (< 1%).

Brightfield and green fluorescence whole‐well images (4× objective) were captured before treatment and 120 h post‐treatment using the Tecan Spark Cyto, maintained at 37 °C with 5% CO_2_. Hoechst 33342 was used as an endpoint viability marker and imaged in the blue channel at 120 h. Cell counts, both label‐free and fluorescent, were performed using Spark Control software. Viability was quantified as the difference between the Total Cell Count (label‐free at 0 h and blue at 120 h) and the Total Death Cell Count (green fluorescence). This was used to calculate the Normalized Growth Rate (NGR) as previously described [20]. Based on the NGR values, drug effects were classified as follows: NGR >1, proliferative effect; NGR = 1, normal growth (similar to vehicle control); NGR = 0, complete growth inhibition; and NGR = −1, complete cell death (similar to positive control). Experiments were performed with 4 technical replicates and at least 2 biological replicates for the CUTO‐37 cell line, and at least one biological replicate for the CUTO‐28 cell line.

### 2.4 Immunoblotting

A total of 5 × 10^5^ cells were seeded in 6‐well plates and treated 24 h later during 72 h with TKIs dissolved in DMSO. Total protein was collected using RIPA lysis buffer (Thermo Fisher Scientific) supplemented with a tablet of PhosSTOP (Roche) and cOmplete (Roche) per 10 mL of buffer. Protein quantification was performed using the Pierce BCA Assay (no.: 23227; Thermo Fisher) and the VICTOR Nivo plate reader (Perkin Elmer) following the manufacturer's instructions. Ten micrograms of total, denatured protein was loaded in a 4–12% PAGE gel (Invitrogen) initially during 10 min at 100 V followed by 50 min at 200 V. Proteins were then transferred to a PVDF membrane, which was afterward blocked with milk powder dissolved in TBST buffer 1.5% (w/v). Eventually, membranes were incubated with 1 : 2000‐diluted primary antibodies anti p‐ROS1 Tyr2274 (no.: 3078; Cell Signaling), anti‐ROS1 (Clone D4D6, no.: 3287; Cell Signaling), anti p‐Erk^1/2^ Thr202/Tyr204 (no.: 4370; Cell Signaling), anti‐Erk^1/2^ (no.: 9102; Cell Signaling) and anti‐GAPDH (no.: 5174; Cell Signaling). As a secondary antibody, HRP‐conjugated goat‐anti‐rabbit was used (no.: 7074; Cell Signaling). Finally, membranes were developed using SuperSignalTM West Femto Maximum Sensitivity Substrate (no.: 34094; Thermo Scientific). The resulting images were quantified using ImageQuant TL 1D version 8.2 (General Electric).

### 2.5 Statistical analysis

A 2‐way ANOVA using GraphPad Prism v8 was used to study differences in AOC_NGR between WT and mutant lines. Dunnett's multiple comparisons test was performed to determine differences between WT and mutant cell lines. The chosen alpha value was α = 0.05. Normality and homocedasticity were assumed to perform the 2‐way ANOVA.

### 2.6 Molecular docking

SMINA was employed to generate docked complexes for ROS1^WT^ and ROS1^mutants^ using default settings. The search space was centered on the gatekeeper residue L2026‐Cβ atom with a 25 Å box size along the x, y, and z axes. PyMol was used to evaluate the docked complexes by comparing them to reference PDB structures: 3ZBF (ROS1 bound with crizotinib), 4CLI (ALK bound with lorlatinib), 4MKC (ALK bound with ceritinib), 5FTO (ALK bound with entrectinib), and 7VKO (TrkA bound with repotrectinib). The docked pose that closely matched the reference structure was chosen for further analysis. These selected docked complexes were subsequently examined using all‐atom MD simulations. Furthermore, a detailed overview of system preparations and production runs is provided in our previous study [26].

## 3 Results

In the absence of experimentally determined activated state of ROS1 kinase harboring mutant positions, we employed homology modelling to generate relevant structure models to investigate the impact of ROS1 kinase domain mutations. We employed a PDB template structure (PDB ID: 3ZBF) known for its classical DFG‐in conformation (activated state), as documented in the literature [30]. We then successfully modelled ROS1 mutations (G2032R at the solvent front, L2026M at gatekeeper position, and S1986Y outside the active site) using PyMOL's mutagenesis wizard. We selected the best side chain rotamers based on their highest probability and minimum clash score. All simulations were stable after the equilibration time and concatenated trajectories of 1.8 μs (ROS1^WT^ and ROS1^mutants^) were analyzed.

### 3.1 Evaluation of ROS1^WT^ ‐TKI docked structures obtained through homology modelling

Molecular docking of the ROS1^WT^ kinase domain using SMINA was successfully performed with ATP‐competitive type I TKIs. Docking was performed for crizotinib, ceritinib, lorlatinib, entrectinib, repotrectinib and ATP as ligand. We compared generated docked complexes with reference experimental structures from PDB (3ZBF; crizotinib, 4MKC; ceritinib, 4CLI; lorlatinib, 5FTO; entrectinib, 7VKO; repotrectinib, and 4GT3; ATP) to determine the optimal binding poses. A detailed overview of three‐dimensional (3D) docked poses for the ROS1^WT^ kinase domain with selected ligands and 2D receptor–ligand interactions is provided in Fig. S1. Optimal docked complexes for ROS1^WT^ and ROS1^mutants^ were selected to perform subsequent MD simulations.

**Fig. 1:**
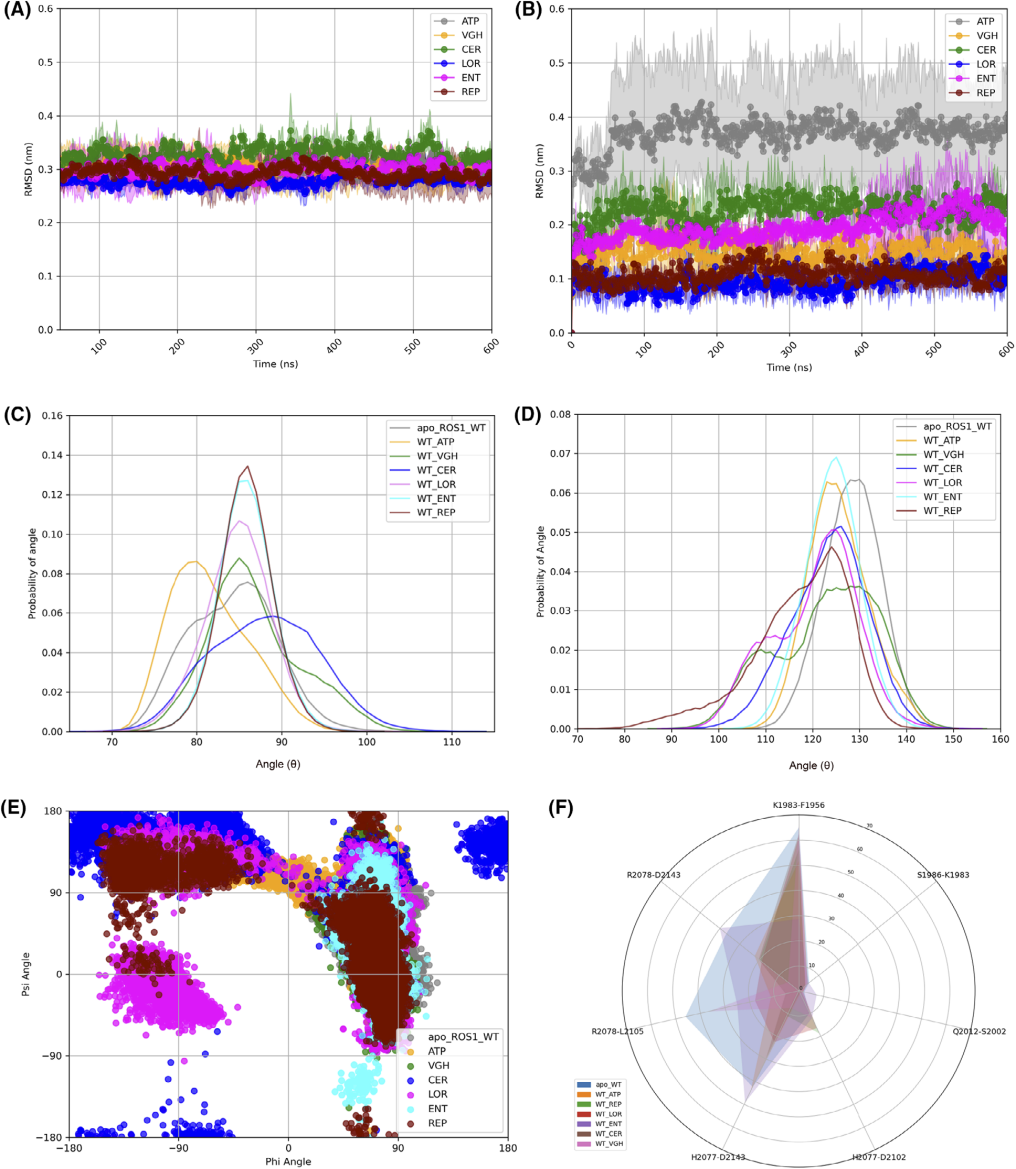
Comparative analysis of the ROS1^WT^ kinase domain using ligand‐bound simulations with ATP and selected type I TKIs. (A) Root mean square deviation (RMSD) comparison for ROS1 kinase domain residues using backbone atoms. (B) RMSD comparison using ligand (heavy atoms) and protein (backbone atoms). (C) Comparison of the dynamics of three‐point angle calculations using residues (1989‐2004‐2145). (D) Comparison of dihedral angle using residues (1982‐1954‐2003‐2112). (E) Ramachandran plot comparisons for residue R2078 from the HRD motif. (F) Qualitative comparison of Hydrogen‐bond profiles using selected interactions for ROS1^WT^ and ROS1^mutants^.

We analyzed protein stability using backbone atoms via root mean square deviation (RMSD) values. The values clustered between 0.25 nm to 0.35 nm, indicating stable and compact models (Fig. 1A).

The preservation of interactions between the ROS1 kinase domain and docked ligands was also assessed using RMSD values calculated between protein backbone and ligand heavy atoms. All ROS1^WT‐ligand^ complexes remained stable during the production run for up to 600 ns in three replicates. Stable binding was observed for all inhibitor compounds with RMSD values below 0.3 nm (Fig. 1B). Notably, ROS1^WT‐ATP^ simulations showed the highest RMSD values, averaging around 0.4 nm with a higher standard deviation (SD) as compared to the other systems.

**Fig. 2:**
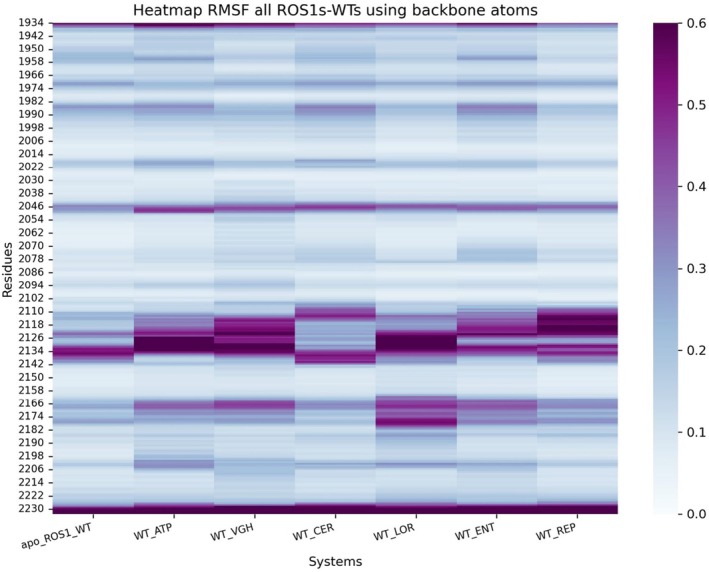
Heatmap illustrates the comparative root mean square fluctuation (RMSF) profiles for the ROS1^WT^ kinase domain in both apo (unbound) and bound simulations with ATP and type I TKIs. The color gradient ranges from 0 Å to 0.6 Å, with darker colors indicating higher flexibility and lighter colors indicating lower flexibility. The x‐axis provides system information, while the y‐axis represents the kinase domain residues.

Root mean square fluctuation (RMSF) comparisons highlighted regions of variable flexibility in both apo (unbound) and bound simulations (Fig. 2).

G‐loop residues between 1950 and 1958 were more flexible in ROS1^WT‐ATP^ and ROS1^WT‐ENT^ simulations. A loop region comprising residue positions 1966 and 1974 (connecting two adjacent β‐strands) showed flexibility in all simulations except for ROS1^WT‐ATP^. Furthermore, a loop region with residues between 2038 and 2046 was hyperflexible in all bound systems, compared with unbound simulations. The DFG motif, present at the start of the activation loop (A‐loop), together with the adjacent residue positions 2102 to 2142, was the most variable region in most simulations. Interestingly, for unbound simulations only the C‐terminal part of the activation loop showed higher flexibility. Finally, residues forming the αG‐helix between 2166 and 2174 exhibited hyperflexibility in all bound simulations.

Furthermore, αC‐helix rotation was assessed through a three‐point angle calculation using the Cα‐atom of residues 1989‐2004‐2145. This analysis demonstrated a consistent profile among type I inhibitors, with a normal distribution topping around 85°. This was in clear contrast with ATP‐bound simulations, which exhibited a smaller angle, peaking around 78°. For further details see Fig. 1C.

Additionally, a dihedral angle analysis using the Cα‐atom of residues 1982‐1954‐2003‐2112 provided insights into the combined dynamics of the G‐loop, αC‐helix, and the A‐loop. Most ligand‐bounded simulations showed a normal distribution pattern, peaking between 120° and 130°. However, crizotinib and lorlatinib simulations displayed a bimodal distribution, indicating additional conformations with a lower dihedral angle between 100° and 110° (Fig. 1D). We examined the residue dynamics of R2078 (within the HRD motif) using Ramachandran analysis, revealing that the conformational states sampled by R2078 differed across apo (unbound), ATP‐bound, and TKI‐bound ROS1^WT^ simulations (Fig. 1E). Additionally, we analyzed the hydrogen bonding network involving residues from the HRD and DFG motifs. Notably, the highest occupancy of interactions between the R2078/L2105 pair was observed in ROS1 WT apo simulations (Fig. 1F).

**Fig. 3:**
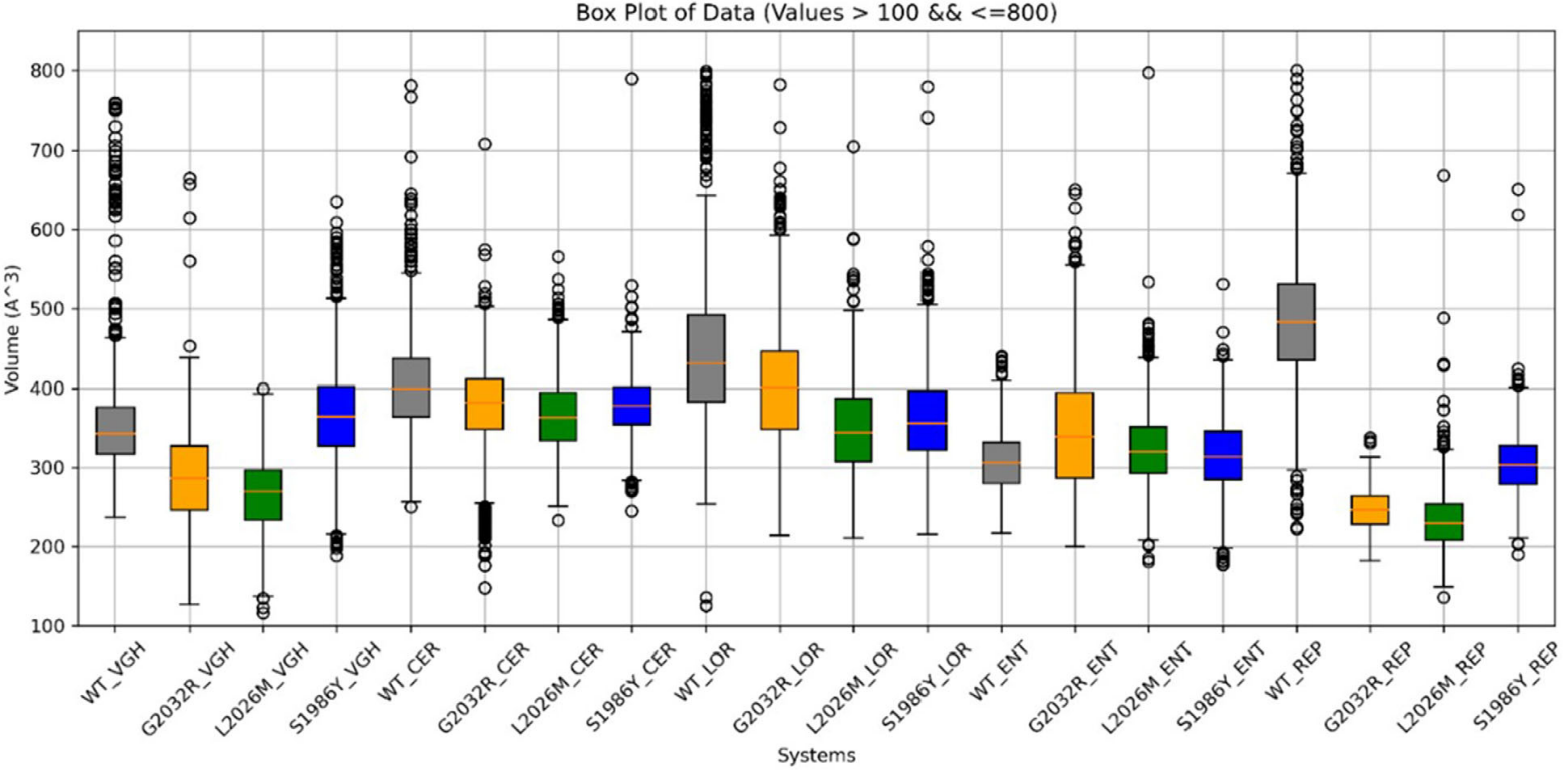
POVME analysis for active site pocket calculations for ROS1^WT^ and ROS1^mutants^ using TKI bounded simulations. ROS1^WT^ systems are represented in gray while ROS1^mutants^ G2032R, L2026M, and S1986Y bound to selected TKIs are shown in orange, green, and blue, respectively. Box‐and‐whisker plots show the distribution of volume values (Å^3^) for each system. The center line in each box represents the median value, while the lower and upper edges of the box indicate the 25th (Q1) and 75th (Q3) percentiles, respectively. Only values between 100 and 800 Å^3^ are included. Differences in box height and median position reflect variation and central tendency among systems.

Moreover, we analyzed the dynamics of active site pocket volume alterations during ligand‐bound simulations by examining concatenated trajectories. Using 1650 frames with a fixed step size, we calculated average pocket sizes for all ROS1^WT^ and ROS1^mutant^ simulations. ROS1^CER‐WT^ exhibited the largest average pocket size at approximately 461Å^3^, while ROS1^ENT‐WT^ and ROS1^WT‐REP^ simulations showed the smallest pocket size at 306Å^3^ and 305Å^3^, respectively. Furthermore, ROS1^VGH‐WT^ and ROS1^LOR‐WT^ presented average values around 343Å^3^ and 400Å^3^, respectively. An overview of these pocket sizes is provided in Fig. 3 and Fig. S8–S12.

### 3.2 ROS1 G2032R, L2026M, and S1986Y mutations impact protein dynamics and crizotinib binding

We compared the stability of ROS1 kinase domain bound to crizotinib in WT and mutant states to understand the potential impact of point mutations (Fig. S2A). Although the difference in overall average RMSD value was not striking, the distribution profiles provided valuable insights. For ROS1^WT^ simulations, more than three conformations with RMSD ranging from 0.1 nm to 0.7 nm were observed, whereas G2032R and L2026M mutations displayed a lower number of conformations. Interestingly, the S1986Y mutation displayed a profile resembling a normal distribution with a single RMSD peak around 0.3 nm. Comparing ligand/protein interaction stability between ROS1^WT^ and ROS1^mutant^ simulations, higher RMSD values were revealed for crizotinib bound to G2032R and L2026M mutants, with all values averaging above 0.16 nm over time. The highest RMSD values were observed for the S1986Y mutant model, averaging around 0.35 nm (Fig. S2B).

Analyzing the ∆RMSF profiles (WT‐mutant) highlighted differences in flexibility between mutant models (Fig. S3). The G‐loop region (residues between 1950 and 1958), for example, was notably more flexible in the case of L2026M and S1986Y mutations as compared to G2032R. Additionally, residues forming loop regions, for example, the loop connecting the initial part of the αC‐helix (residues between 1982 and 1990), were more flexible in the mutation models. Interestingly, the L2026M mutation exhibited multiple regions with increased flexibility containing residues between locations 2014 to 2022, 2038 to 2046, 2070 to 2078, and 2102 to 2110, which was not consistent with G2032R and S1986Y mutations. Furthermore, for G2032R and S1986Y mutations, stabilization in the C‐terminal part of the A‐loop was observed as compared to WT, whereas the G2032R mutation displayed additional hyperflexible regions encompassing residues 2134 to 2142 and 2166 to 2174.

The angle distribution profiles for crizotinib‐bound simulations were similar between ROS1^WT‐VGH^ and ROS1^mutant‐VGH^ simulations, although the width of the distribution differed, especially for L2026M. Dihedral comparisons revealed a distinct shift toward a normal distribution for G2032R and S1986Y mutants, while L2026M retained the WT profile with multiple peaks. Ramachandran analysis revealed additional conformational states characterized by distinct phi/psi angle combinations involving residue R2078 across all crizotinib‐bound ROS1 mutant simulations (Fig. S2E). Moreover, in ROS1^G2032R‐VGH^ simulations, the hydrogen bonding interactions between the residue pairs H2077/D2143 and R2078/D2143 exhibited the highest occupancy (Fig. S2F).

Active site pocket calculations revealed that both G2032R and L2026M displayed a reduced pocket volume, with the latter resulting in the smallest pocket, averaging at 269Å^3^. In contrast, the S1986Y mutation resulted in an increased pocket volume, averaging at 361Å^3^. A comparative overview of crizotinib binding with different mutant ROS1 kinase models is provided in Table 1.

**Table 1:** Effect of crizotinib on different mutant kinases.

Variant	Pocket volume (A^3^)	RMSD distribution pattern (peaks)	∆RMSF WT‐mutant			Hydrogen bonds (%)			α‐Helix rotation (1989–2004‐2145) degree °	Dihedral distribution (1982–1954–2004‐2112) degree °
			G‐loop	αC‐helix	A‐loop	A^a^	B^b^	C^c^		
WT	343	Multi modal	NA	NA	NA	66.53	11.86	5.51	Single/91.5	Bimodal 110/125
G2032R	286 (***)	Bimodal	Similar	High	Low	37.23	23.49	28.51	Single/83.5	Single 125
L2026M	269 (***)	Uni modal	Similar	High	High	48.91	6.60	2.78	Single/89.5	Multiple 98/108/128
S1986Y	361 (***)	Uni modal	High	High	Low	27.54	18.95	34.08	Single/89.0	Single 125

*Note*: *T*‐test significance values between ROS1WT and ROS1mutants are indicated by ***, **, and * symbols. (*P < 0.05, **P < 0.01, ***P < 0.001).

a K1983‐F1956.

b H2077‐D2102.

c R2078‐D2143.

### 3.3 ROS1 G2032R hampers binding to first generation inhibitors

For ceritinib‐bound simulations, analyzing the stability of interactions between protein backbone and ligand heavy atoms revealed the highest RMSD value for ROS1^G2032R‐CER^ simulations with an average value above 0.3 nm and greater SD as compared to other systems, clustering around 0.25 nm (Fig. S4A). The protein RMSD distribution exhibited a normal pattern with a single dominant peak across all systems. Despite the unimodal distribution patterns, the RMSD distributions displayed reduced variance and lower averages in mutants compared with WT (Fig. S4B).

The comparison of ∆RMSF profiles showed a limited impact of mutations on overall kinase flexibility. Notably, the G2032R mutation exhibited higher flexibility for residues 1966 to 1974 as compared to other mutations. Additionally, the N‐terminal part of the A‐loop (starting with the DFG motif) was stabilized in all mutations as compared to WT, while the region containing residues 2118 to 2134 (forming the C‐terminal part of the A‐loop) gained additional flexibility in G2032R and L2026M mutations only. Moreover, the αG‐helix showed added flexibility more specifically in G2032R mutations.

The selected angle distribution profiles between ROS1^WT‐CER^ and ROS1^mutants‐CER^ simulations showed comparable and normally distributed profiles with a single dominant conformation. The mutant simulations exhibited more compact and limited distributions, mostly peaking between 85° to 90°, in contrast to WT which displayed a wider range between 70° to 105° (Fig. S4C). Dihedral angle calculations were mainly in line with WT, except for L2026M, showing a bimodal distribution (Fig. S4D). Ramachandran analysis revealed the widest range of conformational states for residue R2078 in ROS1^WT‐CER^ simulations, while ROS1^G2032R‐CER^ simulations displayed the most compact conformational space (Fig. S4E). Additionally, the occupancy of hydrogen bonding interactions between the R2078/L2105 pair was less than 20% across all systems, except for ROS1^G2032R‐CER^ simulations, where higher occupancy was observed (Fig. S4E).

In addition, pocket size calculations indicated that all mutant ROS1^WT‐CER^ systems exhibited similar, reduced pocket volumes compared with WT, averaging between 361Å^3^ and 381Å^3^.

### 3.4 Improved ligand binding for ROS1^WT^ and ROS1^mutants^ with second generation TKI lorlatinib and entrectinib

Interactions between lorlatinib and the kinase domain remained stable across all systems, with average values clustering around 0.20 nm (Fig. S5A). The stability of the kinase domain was evident from the RMSD distribution profile, which showed a normal distribution pattern for all systems except the S1986F mutant with a bimodal distribution. The protein RMSD values generally ranged between 0.10 nm to 0.60 nm, except for the S1986Y mutation, reaching up to 0.70 nm (Fig. S5B).

As per ∆RMSF comparison, we observed a loop region connecting two β‐strands (residues 2017 and 2022) being more flexible for L2026M and S1986Y mutations as compared to the G2032R. Another region with a loop conformation consisting of residues 2046 to 2054, which is also engaged in connecting two α‐helices, showed slightly higher flexibility for all mutations as compared to the WT. The HRD motif residues showed a slight increase in stability for G2032R and L2026M mutations, unlike S1986Y. Notably, S1986Y simulations exhibited overall increased flexibility across key regions when compared to WT, especially in the A‐loop region along with G‐loop and the αC‐helix. However, the C‐terminal part of A‐loop was stabilized for L2026M mutant as compared to both WT and other mutations (Fig. S3).

The angle distribution profiles showed similar unimodal patterns for ROS1^WT‐LOR^ and ROS1^mutant‐LOR^ simulations, with all systems ranging between 67° to 103°. ROS1 G2032R and L2026M mutant systems showed a slight increase in the peak value, averaging around 86.5°, while S1986Y simulations had a lower peak around 83° compared with WT simulations. Interestingly, despite the similarities in angle distribution, dihedral distribution patterns differed significantly across WT and mutants. Only the L2026M mutation displayed a strong bimodal distribution, whereas the unimodal distribution of the S1986Y simulation was shifted to a lower peak of 115° (Fig. S5C,D). Ramachandran analysis revealed a distinct conformational cluster for residue R2078 in ROS1^WT‐LOR^ simulations, which was absent in all ROS1^mutant‐LOR^ simulations (Fig. S5E). Additionally, the interaction between the R2078/L2105 pair exhibited the highest occurrence in ROS1^WT‐LOR^ simulations (Fig. S5F).

Furthermore, pocket size calculations showed that WT and G2032R systems exhibited similar pocket sizes, while L2026M and S1986Y mutations resulted in reduced pocket volumes of 343Å^3^ and 353Å^3^, respectively.

Similar ligand‐binding profiles were observed for ROS1^WT‐ENT^ and ROS1^mutant‐ENT^ simulations, comparable to those seen with lorlatinib. Interactions between entrectinib and the kinase domain for both WT and mutant states remained stable, with averaged values clustered around 0.25 nm (Fig. S6A). The RMSD distribution pattern affirmed the stability of entrectinib‐bound simulations in both WT and mutant states, with all systems ranging from 0.1 nm to 0.6 nm. Although the distribution range was similar, multiple peaks were observed for the G2032R and L2026M mutations, indicating variable conformations. Despite these additional peaks, the overall stability range was not altered (Fig. S6B).

From the ∆RMSF comparison, we noticed differences for key regions highlighted above. These differences were observed to be mutation specific in these simulations. G2032R mutation showed added flexibility for residues of αC‐helix and the N‐terminal part of the A‐loop, while residues belonging to αG‐helix and the following loop were more stabilized as compared to WT. For L2026M mutation, hyperflexibility was observed in the loop conformation for residues from 1966 to 1974 which are also connecting two adjacent β‐strands. The initial regions of the A‐loop containing the DFG motif were more flexible in the G2032R mutation, while the C‐terminal region of the A‐loop showed greater flexibility in the S1986Y mutant simulation. Additionally, the S1986Y mutant exhibited opposite dynamics in the A‐loop compared with the G2032R simulations.

The angle distribution profiles were similar between ROS1^WT‐ENT^ and ROS1^mutant‐ENT^ simulations, with average peak values clustering between 85° to 90°. However, differences were noted in dihedral distribution patterns where WT simulations exhibited a normal distribution pattern with a single peak around 125°, whereas G2032R and L2026M mutations showed bimodal distribution patterns with additional peaks around 105° and 115°, respectively. Moreover, for S1986Y simulations, no bimodal distribution was observed, but the angle peaked around 115° (Fig. S6C,D). Ramachandran analysis revealed a distinct conformational space for the G2032R mutant in entrectinib‐bound simulations, with unique combinations of phi/psi angles sampled by residue R2078 (Fig. S6E). Additionally, the highest occupancy of interactions was observed between the H2077/D2143 and R2078/D2143 pairs in ROS1^G2032R‐ENT^ simulations (Fig. S6F).

Besides, pocket volume calculations revealed that the G2032R mutation displayed the largest pocket at 337Å^3^, while the smallest pocket was seen for WT 306Å^3^. While for L2026M and S1986Y mutations, the average pocket sizes were calculated at 321Å^3^ and 312Å^3^, respectively.

### 3.5 Comparative analysis of ROS1^WT^ and ROS1^mutant^ kinase domain with next‐generation TKI repotrectinib

Conservation of interactions between repotrectinib and the protein backbone was highlighted with low RMSD values which remained stable across ROS1^WT^ and ROS1^mutant^ simulations. The average values for all systems clustered between 0.1 nm and 0.2 nm (Fig. S7A). The protein backbone RMSD distribution profile exhibited a bimodal pattern for WT simulations, spanning from 0.1 nm to 0.7 nm. Contrastingly, G2032R mutation showed a compact range up to 0.55 nm, with multiple additional peaks. Conversely, the distribution profile for L2026M and S1986Y simulations displayed a unimodal pattern, with a narrow compact RMSD profile ranging between 0.50 nm and 0.50 nm (Fig. S7B).

The comparison of ∆RMSF profile revealed differences across several regions for ROS1 mutants as compared to its WT counterparts. G2032R mutation displayed minor added flexibility in the αC‐helix while the loop conformation for residues between 2038 and 2046 was more stabilized in G2032R and L2026M mutations, contrasting with S1986Y simulations. The region containing the HRD motif showed a similar trend across mutations. Additionally, the N‐terminal part of the A‐loop exhibited hyperflexibility across all mutant simulations, whereas the C‐terminal region was hyperflexible in S1986Y simulations only. The stability of the αG‐helix and the following loop was consistent across all mutant systems in repotrectinib‐bound simulations.

The angle distribution pattern followed a similar scheme across all simulations, with a normal curve and an average single peak around 87°. Despite this similarity, differences in dihedral distribution were evident. ROS1^WT‐REP^ simulations displayed a wide range of values between 75° and 145°, with a dominant peak around 125°, while G2032R and L2026M simulations exhibited a bimodal distribution with an additional peak averaging around 110°. In S1986Y simulations, a normally distributed curve with a single peak value at approximately 128° was observed (Fig. S7C,D). All ROS1^mutant‐REP^ simulations displayed a more compact Ramachandran plot for residue R2078 compared with the ROS1^WT‐REP^ simulations, which exhibited an additional conformational cluster (Fig. S7E). The highest occupancy of interactions between the H2077/D2143 and R2078/D2143 pairs was observed in ROS1^L2026M‐REP^ simulations (Fig. S7F).

Moreover, for pocket size calculations, the WT simulations exhibited the largest pocket at 305Å^3^, while for L2026M mutation the smallest pocket at 228Å^3^ was observed. Additionally, for ROS1 G2032R and S1986Y mutants, the average pocket size was calculated at 246Å^3^ and 302Å^3^, respectively. A comparative overview of repotrectinib binding with different mutant ROS1 kinase models is provided in Table 2.

**Table 2:** Effect of repotrectinib on different mutant kinases.

Variant	Pocket volume (A^3^)	RMSD distribution pattern (peaks)	∆RMSF WT‐mutant			Hydrogen bonds (%)			α‐Helix rotation (1989–2004‐2145) degree °	Dihedral distribution (1982–1954–2004‐2112) degree °
			G‐loop	αC‐helix	A‐loop	A^a^	B^b^	C^c^		
WT	305	Multi modal	NA	NA	NA	56.36	18.77	19.98	Single/85	Single/125
G2032R	246 (***)	Multi modal	Similar	High	Low	44.79	14.23	38.56	Single 84.5	Bimodal/110/125
L2026M	228 (***)	Uni modal	Similar	Similar	Low	59.56	21.85	55.51	Single/85.5	Bimodal 110/130
S1986Y	302 (*)	Uni modal	High	High	Low	49.58	24.64	25.06	Single/85	Single/128

*Note*: *T*‐test significance values between ROS1WT and ROS1mutants are indicated by ***, **, and * symbols. (*P < 0.05, **P < 0.01, ***P < 0.001)

a K1983‐F1956.

b H2077‐D2102.

c R2078‐D2143.

### 3.6 Repotrectinib showed the highest efficacy among TKIs in ROS1^mutant^ CUTO‐28 lines

**Fig. 4:**
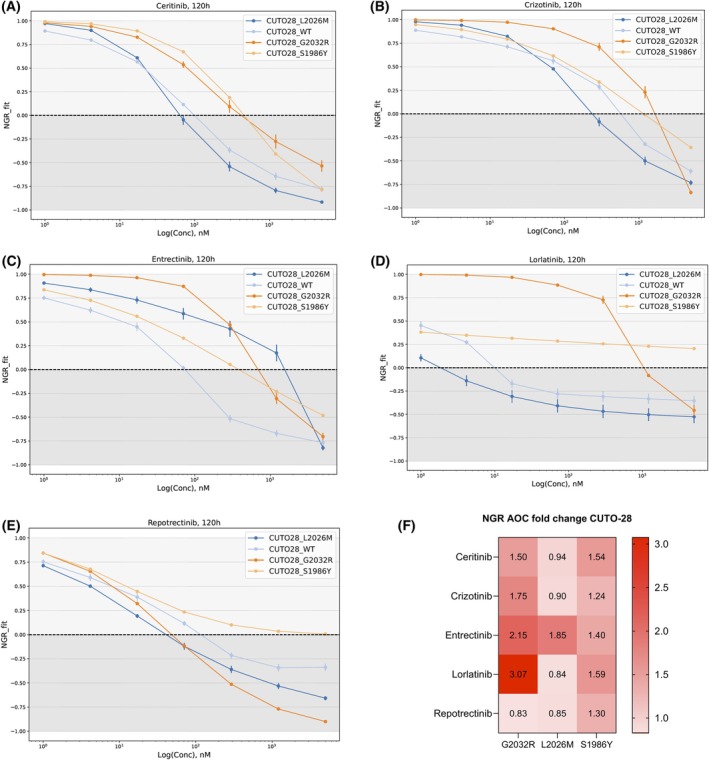
Dose–response curves reflecting the normalized growth rate (NGR ± SEM) of CUTO‐28 lines. TKI treatment with (A) ceritinib, (B) crizotinib, (C) entrectinib, (D) lorlatinib, and (E) repotrectinib; (F) NGR area‐over‐the‐curve (AOC) fold changes in mutant versus WT treated lines. Experiments were performed with four technical replicates and WT (*n* = 3), G2032R (*n* = 2), L2026M (*n* = 2), and S1986Y (*n* = 1) biological replicates.

We then continued with *in vitro* drug assays on genetically engineered patient‐derived cell lines CUTO‐28 and CUTO‐37. Figure 4A–E depicts the dose–response curves of CUTO‐28 expressing ROS1 WT, G2032R, L2026M, or S1986Y treated with ceritinib, crizotinib, entrectinib, lorlatinib, and repotrectinib. To quantify the magnitude of TKI resistance, the fold change in the area‐over‐the‐curve (NGR_AOC FC) in mutant lines versus WT cells was calculated (Fig. 4F).

ROS^G2032R^ cells showed the strongest resistant phenotype across the studied mutations. Repotrectinib was the single compound that successfully induced a cytotoxic effect (FC = 0.83), whereas the remaining TKIs failed to overcome the impact of the G2032R variant. The least effective compound was lorlatinib (FC = 3.07) followed by entrectinib (FC = 2.15), crizotinib (FC = 1.75) and ceritinib (FC = 1.5). Interestingly, CUTO‐28 ROS1^L2026M^ cells were only resistant to entrectinib (FC = 1.85), responding similarly as WT cells to the remaining TKIs. Concerning ROS1^S1986Y^ cells, a refractory effect in a comparable magnitude was observed across all compounds. NGR_AOC fold changes ranged from 1.59 upon lorlatinib treatment, to 1.24 for crizotinib‐treated cells.

**Fig. 5:**
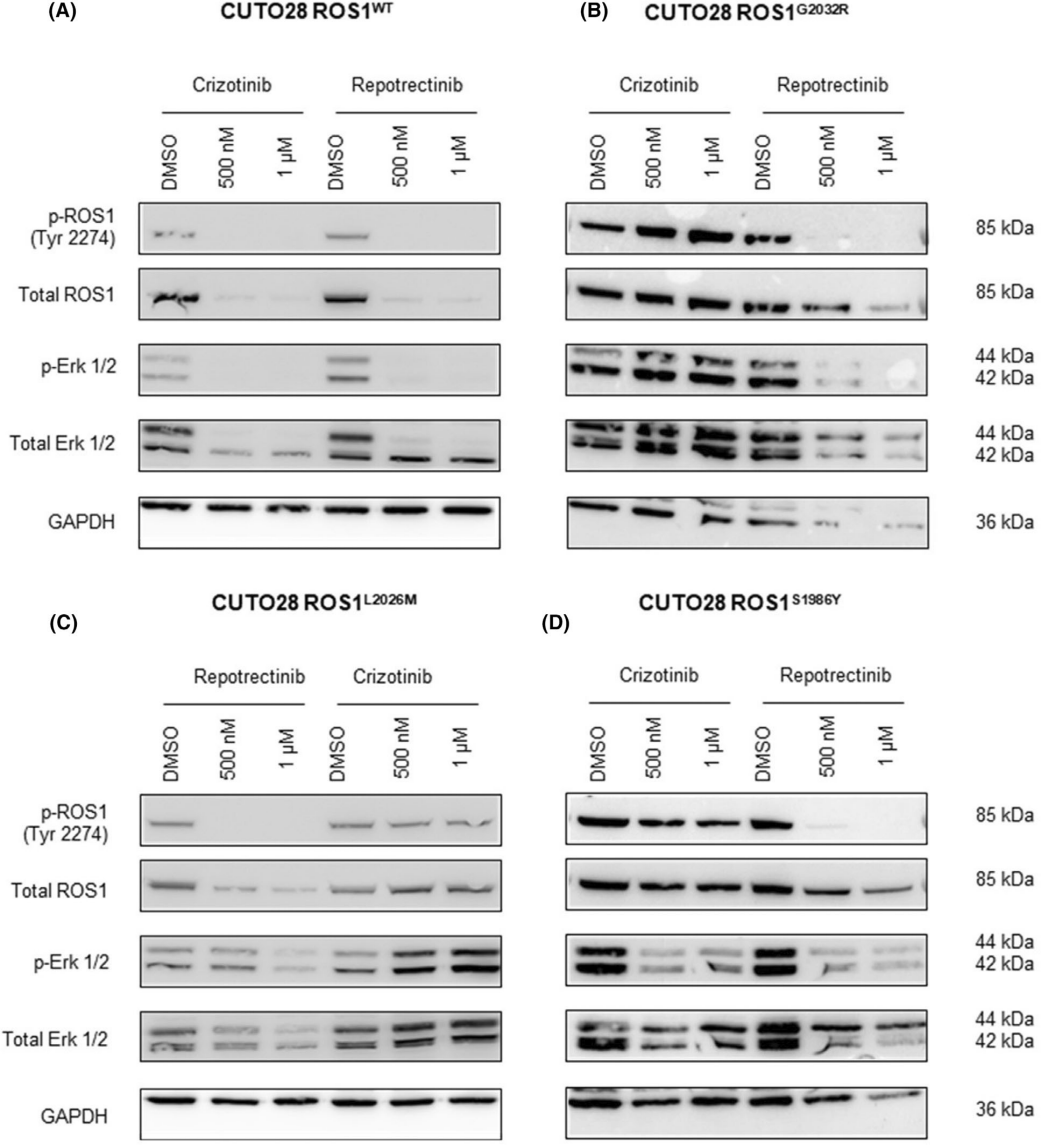
Immunoblotting assay (replicated twice) using crizotinib and repotrectinib‐treated CUTO‐28 cell lines. CUTO‐28 cells are represented as (A) WT, (B) G2032R, (C) L2026M, and (D) S1986Y mutations.

Immunoblotting experiments to accurately determine the levels of phosphorylated ROS1 (Tyr 2274) and Erk^1/2^—one of its surrogate downstream effectors—provided valuable information about the on‐target effects of TKIs. Given that TKIs can inhibit other kinases sharing homology with ROS1, dose–response curves reflect the global cellular response and may therefore include nonspecific inhibition of additional kinases. To specifically assess the direct effect on ROS1, western blotting was performed. Figure 5A shows p‐ROS1 and p‐Erk^1/2^ levels in CUTO‐28 parental cells following treatment with repotrectinib or crizotinib at 500 nm and 1 μm. In the absence of treatment, phosphorylated ROS1 (p‐ROS1) and total ROS1 were readily detected, confirming the ROS1 dependence of this cell line. Treatment with either TKI resulted in complete inhibition of ROS1 phosphorylation, accompanied by inhibition of Erk^1/2^ phosphorylation. Notably, total ROS1 protein levels were also reduced compared with untreated cells. This observation is consistent with inhibitor‐induced destabilization and enhanced turnover of ROS1, likely mediated by engagement of endogenous degradation pathways as reported by Scholes et al [31]. Total Erk^1/2^ levels were modestly reduced, which may represent an indirect downstream consequence of sustained pathway suppression and altered proteostatic regulation following ROS1 inhibition, rather than direct targeting of Erk^1/2^ by these inhibitors.

CUTO‐28 ROS1^G2032R^ cells confirmed the crizotinib‐resistant phenotype, whereas the mutant kinase remained significantly inhibited by repotrectinib at both concentrations (*P* = 0.001 and *P* < 0.001, respectively) (Fig. 5B). These findings indicate that repotrectinib effectively suppresses ROS1 signaling in this resistance context, consistent with its activity against solvent‐front mutations. Similarly, CUTO‐28 ROS1^L2026M^ cells exhibited resistance to crizotinib. Repotrectinib effectively inhibited ROS1 phosphorylation; however, p‐Erk^1/2^ levels were only modestly reduced. This observation suggests that this variant may contribute to partial maintenance of MAPK pathway signaling despite ROS1 inhibition, potentially through altered signaling dynamics or engagement of compensatory mechanisms (Fig. 5C). Finally, CUTO‐28 ROS1^S1986Y^ cells displayed a distinct response to crizotinib, as p‐ROS1 levels remained sustained at both 500 nm and 1 μm, while Erk^1/2^ phosphorylation was only partially inhibited. This dissociation between ROS1 and downstream MAPK inhibition suggests that the S1986Y variant may promote resistance through altered kinase regulation or signaling output. In contrast, repotrectinib effectively inhibited ROS1 phosphorylation in these cells, demonstrating its ability to overcome the resistance conferred by this variant (Fig. 5D). Quantification of p‐ROS1 band intensities across treated cell lines was performed independently for each blot following normalization to GAPDH, and fold‐change values were calculated relative to the corresponding WT control processed in parallel. Given that WT vs mutant densiometric assays were done comparing membranes developed in independent experimental rounds, hence the conclusion that can be drawn are essentially qualitative. The resulting densitometric analysis, intended as a supportive measure and interpreted within the limitations of cross‐blot comparisons, is shown in Fig. S14A.

### 3.7 Repotrectinib elicits the strongest inhibitory effect among TKIs in ROS1^mutant^ CUTO‐37 lines

**Fig. 6:**
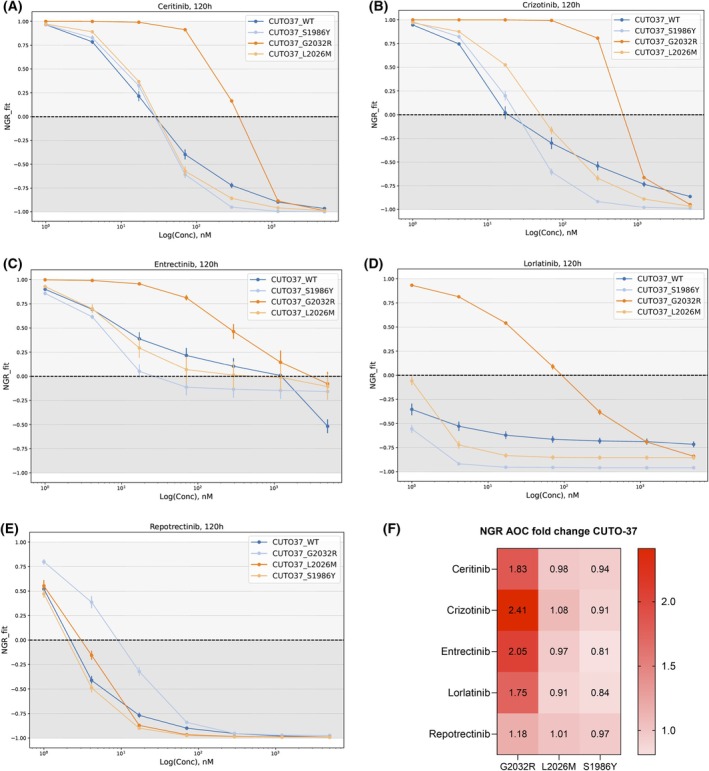
Dose–response curves reflecting the normalized growth rate (NGR ± SEM) of CUTO‐37 lines. TKI treatment with (A) ceritinib (B) crizotinib (C) entrectinib (D) lorlatinib and (E) repotrectinib. (F) NGR area‐over‐the‐curve (AOC) fold changes in mutant versus wild‐type treated lines. Experiments were performed with 4 technical replicates and WT (*n* = 4), G2032R (*n* = 2), L2026M (*n* = 2), and S1986Y (*n* = 3) biological replicates.

In the case of CUTO‐37 cells, G2032R clearly prompted TKI resistance toward all tested compounds (Fig. 6A–E) except for repotrectinib (FC = 1.18) as shown in Fig. 6F. Crizotinib (FC = 2.41) and entrectinib (FC = 2.05) elicited the weakest inhibitory effect, followed by ceritinib (FC = 1.83) and lorlatinib (FC = 1.75). However, as opposed to CUTO‐28 cells, L2026M and S1986Y variants resulted in similar drug‐response profiles to WT cells, depicting AOC fold changes around 1. Figure S13 shows the bar plots summarizing the AOC for every cell line, where no significant differences between WT and mutant lines were detected.

**Fig. 7:**
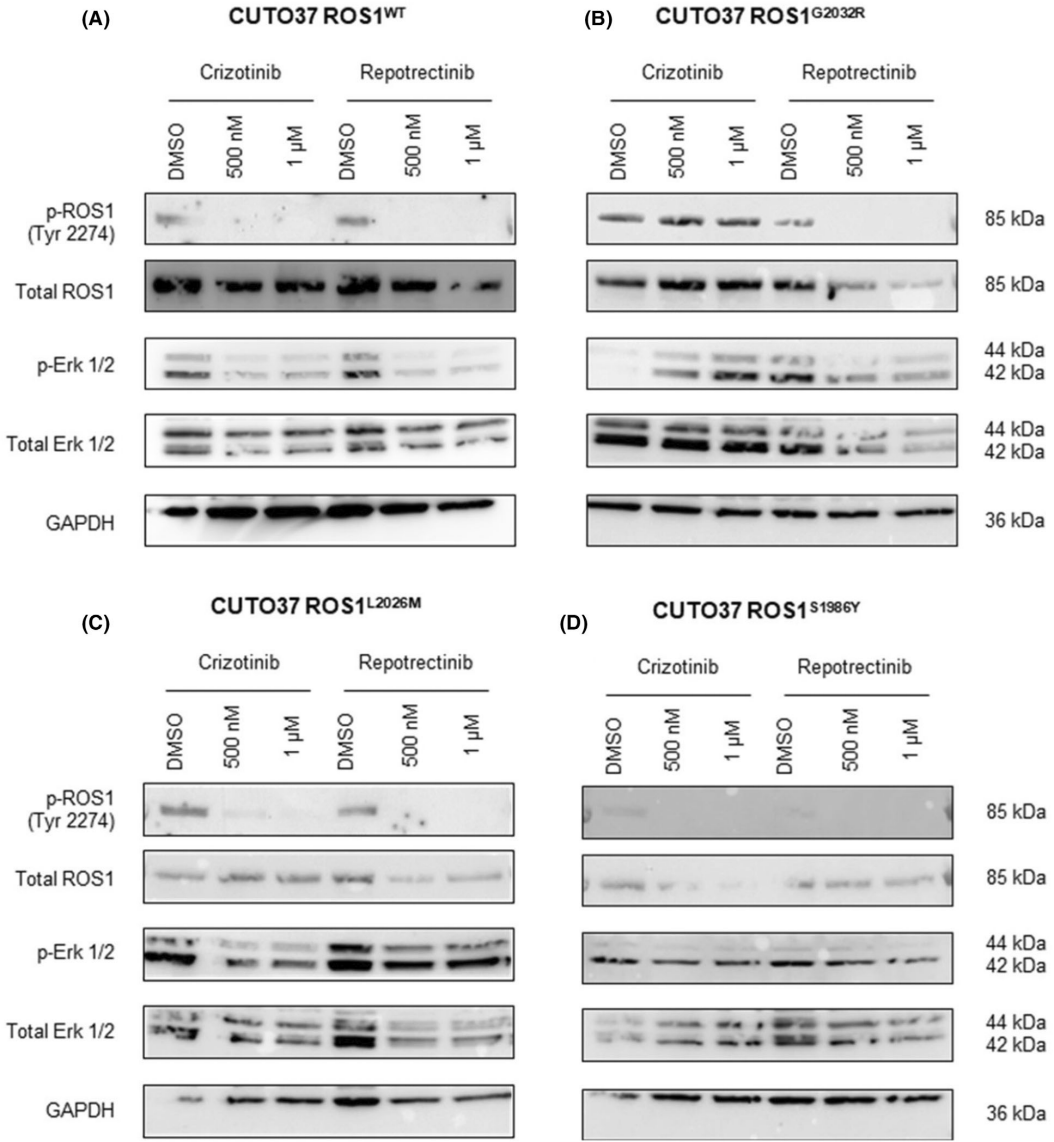
Immunoblotting assay (replicated twice) using crizotinib and repotrectinib‐treated CUTO‐37 cell lines. CUTO‐37 cells represented as (A) WT (B) G2032R and (C) L2026M and (D) S1986Y mutations.

In CUTO‐37 cells, p‐ROS1 levels were maintained only in crizotinib‐treated G2032R cells. A strong inhibition was observed under repotrectinib treatment and in both TKI treatments in the remaining genotypes (Fig. 7A–D). Notably, ROS1^S1986Y^ CUTO‐37 cells exhibited a relatively weak basal p‐ROS1 signal relative to total ROS1. This observation was derived from qualitative comparison across independent blots and therefore does not permit direct quantitative assessment of basal phosphorylation levels between cell lines. Nonetheless, the consistently reduced detectable p‐ROS1 signal contrasts with previous reports describing the S1986Y variant as enhancing autophosphorylation of the ROS1 kinase domain [32]. A summary of basal p‐ROS1 levels in CUTO‐37 cell lines is provided in Fig. S14B. Taken together with the findings in the CUTO‐28 model, these results suggest that the functional impact of resistance‐associated ROS1 mutations may be influenced by the specific ROS1 fusion context.

## 4 Discussion

ROS1 kinase fusions, present in 1–2% of newly diagnosed NSCLC cases, drive tumor progression via oncogenic fusion proteins [33]. These fusions, involving the ROS1 kinase domain with various other genes, result in an oncogenic product with aberrant kinase activity [34, 35]. Targeted therapy with TKIs, including first generation compounds like crizotinib and improved next‐generation TKIs like ceritinib, lorlatinib, entrectinib, and repotrectinib, have shown significant efficacy in NSCLC patients harboring ROS1 kinase in WT and in the case of common ATP‐pocket mutations, leading to improved outcomes [36, 37, 38, 39, 40].

However, the success of targeted therapies is reduced by emerging and more potent point mutations in the ROS1 kinase domain. These mutations have potential implications on drug binding and alter kinase activity, leading to reduced therapeutic response and acquired resistance [41]. Therefore, understanding the impact of these mutations on drug efficacy is crucial for developing effective treatment strategies and decision making while choosing the TKIs and overcoming resistance mechanisms in NSCLC patients [25, 42, 43].

The current relevant patient‐derived ROS1+ NSCLC experimental models are relatively limited, therefore precluding the study of crucial biological insights that define tumor biology and modulate the response to TKIs. Cell‐based models are represented by HCC78, CUTO, and ADK‐VR2 patient‐derived lines [19, 44, 45]. Nonetheless, none of them harbor point mutations within the ROS1 kinase domain which induce intrinsic TKI resistance. Consequently, in this study, we assessed the impact of three variants, G2032R and L2026M located in the active site, and S1986Y which is an allosteric nonactive site mutation, by introducing them in CUTO‐28 and CUTO‐37 lines. It is worth noting that the oncogenic driver rearrangement is TPM3‐ROS1 and CD74‐ROS1, respectively, for the two lines. Hence, the effect of G2032R, L2026M, and S1986Y was modelled in two different genetic backgrounds. Furthermore, no structural data are available for either of these ROS1 mutations.

The invaluable role of computational modelling in advancing research on rare cancers and emerging kinase targets, particularly when experimental structural data is limited, cannot be ignored [46, 47, 48]. In the case of the ROS1 kinase, the lack of sufficient experimental structural information makes computational modelling an essential tool for understanding its structure and dynamics. By employing homology modelling and molecular docking techniques, we have proposed a comparative overview of the ROS1 kinase domain in complex with a series of type I TKIs. This approach allows us to explore the interactions between ROS1 and these inhibitors, even in the absence of direct experimental evidence. We successfully utilized homology modelling to construct ROS1^G2032R^, ROS1^L2026M^ and ROS1^S1986Y^ models in the DFG‐in (active) state. Subsequent docking of ROS1^WT^ models with type I TKIs such as crizotinib, ceritinib, lorlatinib, entrectinib, and repotrectinib was used as a check point for the validation of starting complexes which were subjected to all‐atom MD simulations. We generated nearly identical docked poses for these TKIs with ROS1 as compared to their reference PDB structures. Furthermore, the stability of these docked complexes and receptor–ligand interactions was investigated using protein and ligand RMSDs. The low and compact range for RMSD values in both unbound and ligand‐bound simulations revealed system stability and compact nature of these docked complexes. The aforementioned angle and dihedral calculations provided valuable insights involving different regions of ROS1 kinase to cater ligand binding and potential implications of point mutations on overall ROS1 kinase dynamics. Interestingly, the global protein dynamics also incurred active site variations.

The pocket volume calculations were vital in demonstrating how the mutations and the presence of various ligands affect the volume which can directly dictate inhibitor binding. Significant differences were observed between ROS1 apo and ligand‐bound simulations, with variable hydrogen bonding interactions identified between residue H2077/D2143, R2078/L2105, and R2078/D2143 pairs. These differences underscore the critical role of these interactions in kinase regulation and the mechanism of TKI inhibition, where the presence of a TKI disrupts these interactions. The functional significance of these residues warrants further investigation and experimental validation to elucidate their precise role in kinase regulation and inhibitor interactions.

To complement our in silico investigation with experimental validation, we explored these selected mutations experimentally using patient‐derived cell‐line models. This approach allowed us to mimic clinical activity and generate comparable drug‐response profiles, providing a more comprehensive understanding of how these mutations behave in a clinical context. The drug assays carried out in the newly established ROS1^mutant^ patient‐derived cell lines are aligned with the observations we previously reported in HCC78 cells [20, 49].

ROS1 G2032R mutation has been the most frequently occurring mutation in the patients with a diverse resistance profile against a variety of type I TKIs. This active resistant profile for G2032R mutant can be extrapolated to the p‐ROS1 levels determined in the immunoblotting assays. Both CUTO‐37 and CUTO‐28 ROS1^G2032R^ lines depicted active kinase in the presence of crizotinib at 500 nm and 1 μm. Computational analysis revealed unstable interactions between G2032R and various TKIs, as evidenced by higher ligand RMSD values. This finding aligns with the experimental results showing a broad resistance profile for G2032R against multiple type I TKIs. The low AOC values observed in drug assays for ROS1^G2032R^ lines corroborate the computational predictions of reduced drug efficacy. Loss of conserved interactions between the protein backbone and ligand heavy atoms can be inferred from higher ligand RMSD values for crizotinib bound to ROS1 mutants, which hints toward altered ligand binding, suggesting evidence of TKI resistance.

All the ROS1 mutants showed ligand RMSD values greater than WT, and interestingly, the same observation was true for the nonactive site mutation S1986Y. These differences in ligand RMSD are also in line with our previous study where we compared crizotinib and lorlatinib binding against mutation hotspots 1982 and 1986 [26]. The ∆RMSF analysis revealed that mutations in close proximity can have different consequences on protein flexibility. This was evident in the case of ROS1 TKI‐bound simulations where G2032R and L2026M mutations exhibited multiple differences in G‐loop and the A‐loop dynamics despite residing close to each other in the structure. For allosteric mutations such as S1986Y, we speculate a more complex mechanism of resistance involving differences in active site interactions, conformational changes involving substrate recognition site encompassing the αG‐helix, G‐loop, and the A‐loop conformation [50]. Flexibility analysis using RMSF calculation also yielded consistent highlights. Key kinase domain regions highlighted areas of flexibility and conformational changes, notably involving the G‐loop, A‐loop, and αG‐helix. These observations are in line with previous literature reports [16, 27] and also complement our previous study [26].

Our computational analysis indicated that the S1986Y mutant has varied implications. Differences in ligand binding were observed only with crizotinib; all other ligands displayed steady RMSD profiles comparable to their WT counterparts. Additionally, when comparing crizotinib and repotrectinib simulations, we found that WT protein dynamics exhibited a multimodal distribution, while the S1986Y mutation shifted to a unimodal distribution (Tables 1 and 2).

This suggests that the protein undergoes conformational changes, resulting in an equilibrium shift to a single dominant state. This conformational shift might limit the efficacy of TKIs and enhance the kinase activity of the S1986Y mutant. Additionally, pocket size calculations for repotrectinib‐bound simulations revealed varying sizes across mutations, with WT having the largest pocket and S1986Y displaying the smallest, indicating structural alterations in the binding pocket due to mutations. Despite these changes, repotrectinib binding was not altered, suggesting how it is bypassing the effects of conformational changes and still able to bind the kinase. We can speculate that, given the alteration in the pocket volume, TKIs fail to target the active site effectively while the ATP molecules, which are relatively smaller than these drugs, continue to bind and sustain the overactivation of aberrant kinase.

Current MD methods are constrained by computational limitations, restricting their focus primarily to the kinase domain and making it challenging to capture the broader impact of fusion partners. Alternative approaches such as coarse‐grained modelling, including the MARTINI force field [51, 52], offer an intriguing avenue for further exploration. These methods allow for simulations of larger systems over extended time scales, potentially providing deeper insights into fusion partner effects. Additionally, AI‐driven structure and property prediction tools like ESMFold [53], which rely solely on sequence information, provide an exciting alternate computational exploration. By integrating these approaches, a global understanding of how fusion partners influence kinase dynamics can yield valuable insights.

Regarding ROS1^L2026M^ lines, an evident bypassing of crizotinib‐mediated inhibition was exclusively observed in CUTO‐28 cells. Importantly, in CUTO‐28 cells, despite a total repotrectinib‐mediated inhibition, p‐Erk^1/2^ levels were still detected. Thus, indicating the activation of the MAP kinase pathway in a ROS1‐independent manner. This suggests either the mediation of apoptosis induced by this pathway, a negative regulation feedback mechanism, or the existence of additional interactions between the mutant kinase and other proteins whose effect converges in MAPK pathway activation. The latter mechanism is compatible with the enhancement of the autophagy cell machinery reported in CD74‐ROS1^L2026M^ cells [54, 55]. Computational analysis of the L2026M mutation revealed protein conformational changes, particularly in the dihedral distribution when bound to repotrectinib. MD trajectories indicated stable interactions between repotrectinib and ROS1, even in the presence of mutations. This was supported by experimental data showing strong kinase inhibition by repotrectinib in both WT and mutant cell lines. Repotrectinib exerts its potent effect by stabilizing key interactions between the ligand and the ROS1 receptor, promoting favorable αC‐helix dynamics that mitigate the effects of the mutation, allowing the drug to maintain its inhibitory action.

Experimentally, out of the three studied variants, S1986Y induced the poorest TKI‐resistant activity in both cell lines across compounds, except for crizotinib in CUTO‐28 cells. Such results contrast with the sensitivity to crizotinib observed in CUTO‐37 and HCC78 ROS1^S1986Y^ cells, indicating that the ROS1 fusion partner might play a key role in the resulting kinase conformation. Alternatively, the lack of TKI resistance observed in CUTO‐37 L2026M and S1986Y cells could be a dosage issue, by which not enough CD74‐ROS1^L2026M^ and CD74‐ROS1^S1986Y^ mRNA copies are able to overcome the effect of TKIs. By sequencing the ROS1 rearranged alleles through nested PCR, we determined the presence of more than one ROS1 rearranged alleles in CUTO37 L2026M and S1986Y lines and demonstrated successful editing of approximately half of these alleles (Fig. S14A,B). This indicates sufficient expression of the resistant allele, further pointing toward fusion‐partner‐specific conformations influencing the resistance mechanism. Nonetheless, this hypothesis requires further evidence and experimental validation, particularly in the downstream effectors of the MAPK pathway, which represents a limitation of the study. A relevant next step to be addressed is to determine the impact of the ROS1 mutant load expression. Although the expression of a single ROS1 mutant allele seems to be sufficient to drive TKI resistance, little is known whether tumor cells could benefit from expressing multiple ROS1 mutant alleles or if the strong activation of the MAPK kinase pathway would have a detrimental effect by exhausting the cellular fitness. However, this study couples both *in silico* and *in vitro* methodologies as a proof‐of‐concept to dissect the impact of TKI resistance‐conferring ROS1 kinase domain point mutations.

This study meticulously examined three critical mutations—G2032R, L2026M, and S1986Y—using an innovative approach that combined advanced computational techniques with patient‐derived cell‐line models. Computational modelling played a crucial role in understanding the structural dynamics of ROS1 kinase, employing homology modelling and molecular docking techniques to investigate interactions with various TKIs in the absence of experimental data. This study highlighted how specific mutations can dramatically alter protein flexibility, binding pocket volume, and drug interactions. Notably, the G2032R mutation demonstrated the most significant resistance profile, showing unstable interactions with multiple TKIs and active kinase persistence even in the presence of inhibitors.

An important observation in our study is the reduction of total ROS1 protein levels following treatment with both crizotinib and repotrectinib. This effect is consistent with the concept of inhibitor‐induced kinase destabilization, as recently described by Scholes et al. [31], where small‐molecule inhibitors can promote progressive engagement of endogenous proteolytic pathways, leading to accelerated turnover of their targets. In our system, ROS1 degradation is not an immediate consequence of inhibitor binding, but rather reflects cumulative destabilization over extended treatment, which is captured at the 72 h time point. Importantly, this decrease in total ROS1 complements the observed suppression of phosphorylation, reinforcing the mechanistic link between sustained target engagement, protein destabilization, and downstream signaling inhibition. While short‐term treatments primarily reveal acute kinase inhibition, the observed reduction in total ROS1 underscores the broader functional consequences of prolonged inhibitor exposure, integrating both catalytic inhibition and degradation in the modulation of oncogenic signaling.

Experimental validation using patient‐derived cell lines corroborated the computational predictions, revealing nuanced resistance mechanisms. The L2026M mutation, for instance, demonstrated an ability to bypass crizotinib‐mediated inhibition, while repotrectinib showed promise in maintaining strong kinase inhibition across different mutation profiles. Particularly intriguing was the observation of ROS1‐independent MAP kinase pathway activation, suggesting complex resistance mechanisms that extend beyond direct kinase interactions.

Furthermore, this study underscores the critical importance of personalized treatment strategies, demonstrating how computational and experimental approaches can work synergistically to unravel the intricate molecular mechanisms underlying drug resistance in NSCLC. By providing detailed insights into mutation‐specific responses, this research offers valuable guidance for developing precise and effective therapeutic interventions.

## 5 Conclusions

A comprehensive analysis of ROS1 kinase domain bound to crizotinib, ceritinib, lorlatinib, entrectinib, and repotrectinib yielded consistent conclusions regarding kinase stability and structural dynamics. Interactions between inhibitors and kinase domains in WT state remained stable, although slight differences were observed for selected point mutations, especially for crizotinib and ceritinib. Although the flexibility analysis revealed localized changes in specific regions due to mutations, overall stability was maintained. Furthermore, angle and dihedral distributions were consistent across all simulations, suggesting a similar binding mode for these inhibitors. Whereas in mutant simulations, we observed changes in angle and dihedral distributions, suggesting their role in conferencing resistance.

Moreover, our results highlight the important role of the ROS1 fusion partner in modulating the impact of the different point mutations, revealing a complex landscape of multiple conformations. Although G2032R and L2026M mutations are located within the active site, their role might not be limited to altered ligand binding only. Modelling these three variants in two different patient‐derived cell lines unveiled potential ROS1 fusion‐dependent differences in TKI sensitivity. Consistently with the clinical observations in patients, G2032R represents the most aggressive variant, and S1986Y should be further explored in a TPM3‐ROS1 context to determine its particularly strong effect as opposed to CD74‐ROS1^S1986Y^. Overall, these findings underscore the intricate relationship between mutations and protein dynamics, providing valuable insights that can inform drug design strategies and deepen our understanding of molecular mechanisms underlying diseases.

## Conflict of interest

CD is co‐founder of Orbits® Oncology, an image and data analysis platform that was used in this study.

## Author contributions

GV, GVC, and KODB contributed to the conceptualization, funding acquisition, and supervision. Regarding methodology, FU conducted protein modelling, molecular simulations, and data analysis. MT performed CRISPR/Cas9‐mediated mutagenesis, drug screenings, western blotting, and data analysis. CD contributed to drug screenings and data analysis. FRF and AD executed drug assays and western blotting; and AS assisted with western blotting. FU, MT contributed to the writing – original draft. GV, GVC, KODB, and CD. contributed to the reviewing and editing of the original draft. FU and MT contributed equally to this work.

## Supporting information


**Fig. S1.** ROS1^WT^ kinase domain is shown in a cartoon format, with α‐helices in yellow, β‐sheets in cyan, and loop regions in gray.
**Fig. S2.** Comparative analysis of the ROS1^WT^ and ROS1^mutant^ kinase domain using crizotinib‐bound simulations.
**Fig. S3.** ∆RMSF profiles (WT‐mutant) highlighted differences in flexibility between mutant models.
**Fig. S4.** Comparative analysis of the ROS1^WT^ and ROS1^mutant^ kinase domain using ceretinib‐bound simulations.
**Fig. S5.** Comparative analysis of the ROS1^WT^ and ROS1^mutant^ kinase domain using lorlatinib‐bound simulations.
**Fig. S6.** Comparative analysis of the ROS1^WT^ and ROS1^mutant^ kinase domain using entrectinib‐bound simulations.
**Fig. S7.** Comparative analysis of the ROS1^WT^ and ROS1^mutant^ kinase domain using repotrectinib‐bound simulations.
**Fig. S8.** POVME analysis was conducted to calculate the active site pocket for ROS1^WT^ and ROS1^mutants^ using crizotinib‐bound simulations.
**Fig. S9.** POVME analysis was conducted to calculate the active site pocket for ROS1^WT^ and ROS1^mutants^ using ceretinib‐bound simulations.
**Fig. S10.** POVME analysis was conducted to calculate the active site pocket for ROS1^WT^ and ROS1^mutants^ using lorlatinib‐bound simulations.
**Fig. S11.** POVME analysis was conducted to calculate the active site pocket for ROS1^WT^ and ROS1^mutants^ using entrectinib‐bound simulations.
**Fig. S12.** POVME analysis was conducted to calculate the active site pocket for ROS1^WT^ and ROS1^mutants^ using repotrectinib‐bound simulations.
**Fig. S13.** Drug response across ROS1 variants in CUTO cell lines.
**Fig. S14.** Effect of ROS1 mutations on p‐ROS1 levels following drug treatment.
**Fig. S15.** Validation of engineered CUTO‐37 mutant cell lines.

## Data Availability

The data that support the findings of this study are available in the figures and the Supporting Information of this article.
