# Supplementary material for: In vitro and in silico modelling of ROS1‐positive non‐small cell lung cancer reveals fusion‐dependent tyrosine kinase inhibitor responses
Source: Mol Oncol. 2026 Jun 23:10.1002/1878-0261.70291. Online ahead of print. doi: 10.1002/1878-0261.70291 (PMC13398611; doi:10.1002/1878-0261.70291)
Supplement: Supplementary file 1 — Fig. S1. ROS1WT kinase domain is shown in a cartoon format, with α‐helices in yellow, β‐sheets in cyan, and loop regions in gray. Fig. S2. Comparative analysis of the ROS1WT and ROS1mutant kinase domain using crizotinib‐bound simulations. Fig. S3. ∆RMSF profiles (WT‐mutant) highlighted differences in flexibility between mutant models. Fig. S4. Comparative analysis of the ROS1WT and ROS1mutant kinase domain using ceretinib‐bound simulations. Fig. S5. Comparative analysis of the ROS1WT and ROS1mutant kinase domain using lorlatinib‐bound simulations. Fig. S6. Comparative analysis of the ROS1WT and ROS1mutant kinase domain using entrectinib‐bound simulations. Fig. S7. Comparative analysis of the ROS1WT and ROS1mutant kinase domain using repotrectinib‐bound simulations. Fig. S8. POVME analysis was conducted to calculate the active site pocket for ROS1WT and ROS1mutants using crizotinib‐bound simulations. Fig. S9. POVME analysis was conducted to calculate the active site pocket for ROS1WT and ROS1mutants using ceretinib‐bound simulations. Fig. S10. POVME analysis was conducted to calculate the active site pocket for ROS1WT and ROS1mutants using lorlatinib‐bound simulations. Fig. S11. POVME analysis was conducted to calculate the active site pocket for ROS1WT and ROS1mutants using entrectinib‐bound simulations. Fig. S12. POVME analysis was conducted to calculate the active site pocket for ROS1WT and ROS1mutants using repotrectinib‐bound simulations. Fig. S13. Drug response across ROS1 variants in CUTO cell lines. Fig. S14. Effect of ROS1 mutations on p‐ROS1 levels following drug treatment. Fig. S15. Validation of engineered CUTO‐37 mutant cell lines. [file MOL2-9999-0-s001.pdf]

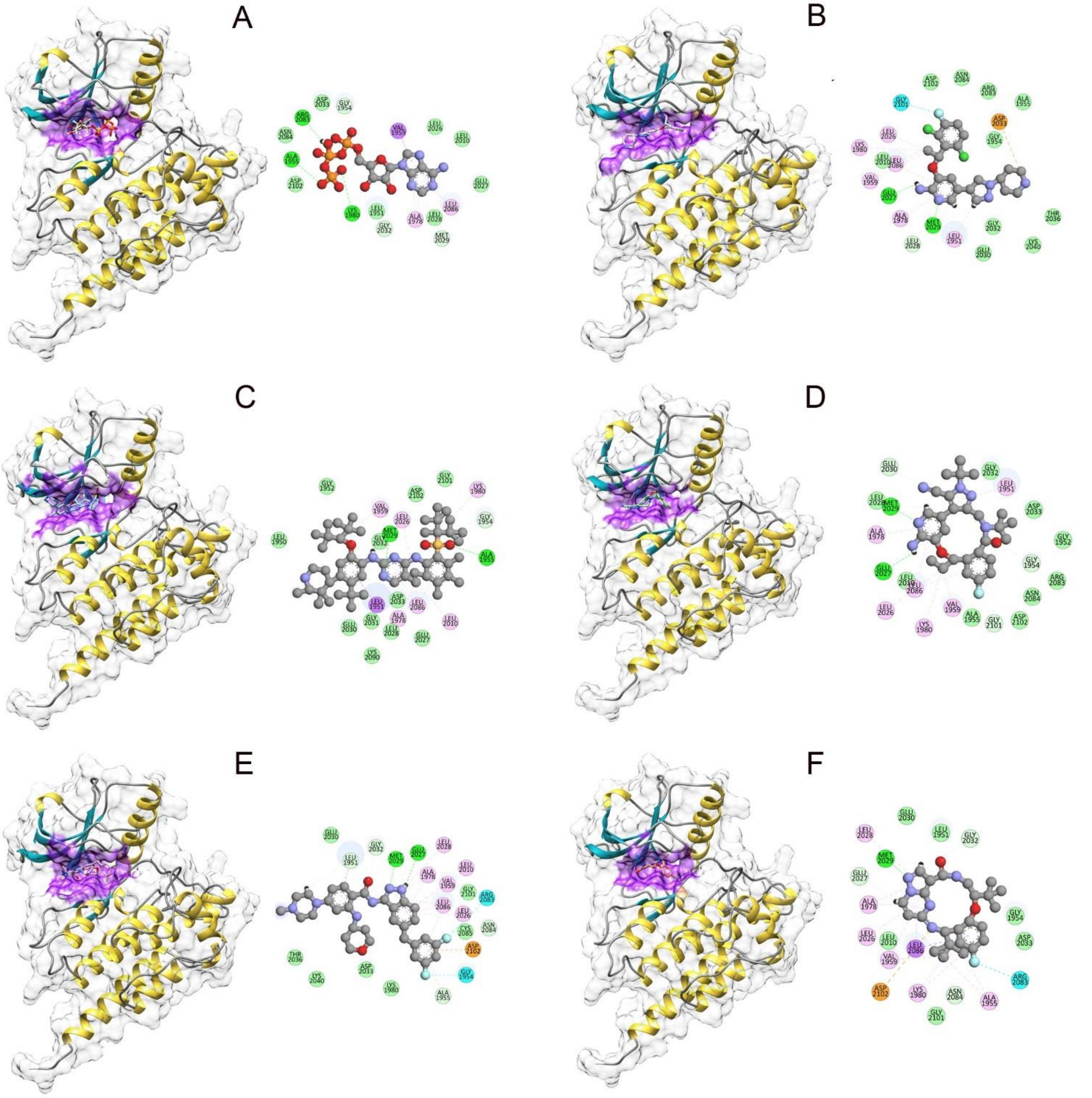

Supplementary Figure 1 The $ROS1^{WT}$ kinase domain is shown in a cartoon format, with α-helices in yellow, β-sheets in cyan, and loop regions in grey. The region within 4Å of the ligand is displayed as a magenta surface. Each panel presents the 3D and 2D orientation of the ligand within the active site pocket. Panels A to F display the docked complexes of selected ligands with the $ROS1^{WT}$ kinase domain: (A) *ROS1-ATP,* (B) *ROS1-VGH,* (C) *ROS1-CER,* (D) *ROS1-LOR,* (E) *ROS1-ENT,* (F) *ROS1-REP.*

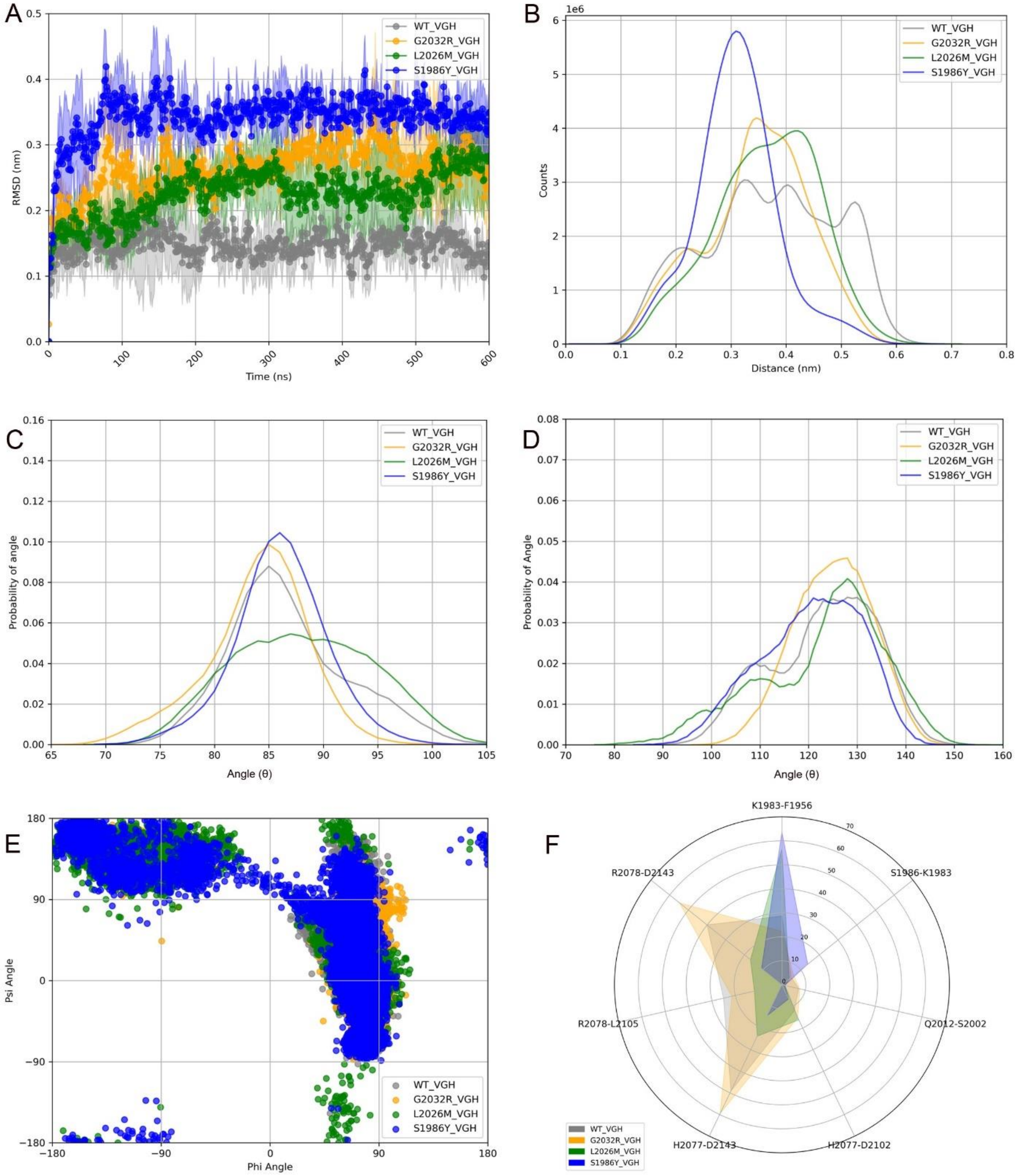

Supplementary Figure 2 Illustrates a comparative analysis of the ROS1^{WT} and ROS1^{mutant} kinase domain using crizotinib-bound simulations. (A) *RMSD comparison for ROS1 kinase domain residues using backbone atoms.* (B) *RMSD comparison for selected ligands (heavy atoms) and protein (backbone atoms).* (C) *Comparison of the dynamics of three-point angle calculations using residues (1989-2004-2145).* (D) *Comparison of dihedral angle using residues (1982-1954-2003-*

2112). (E) *Ramachandran plot comparisons for residue R2078 from the HRD motif.* (F) *Comparison of Hydrogen-bond profiles using selected interactions for ROS1^{WT} and ROS1^{mutants}.*

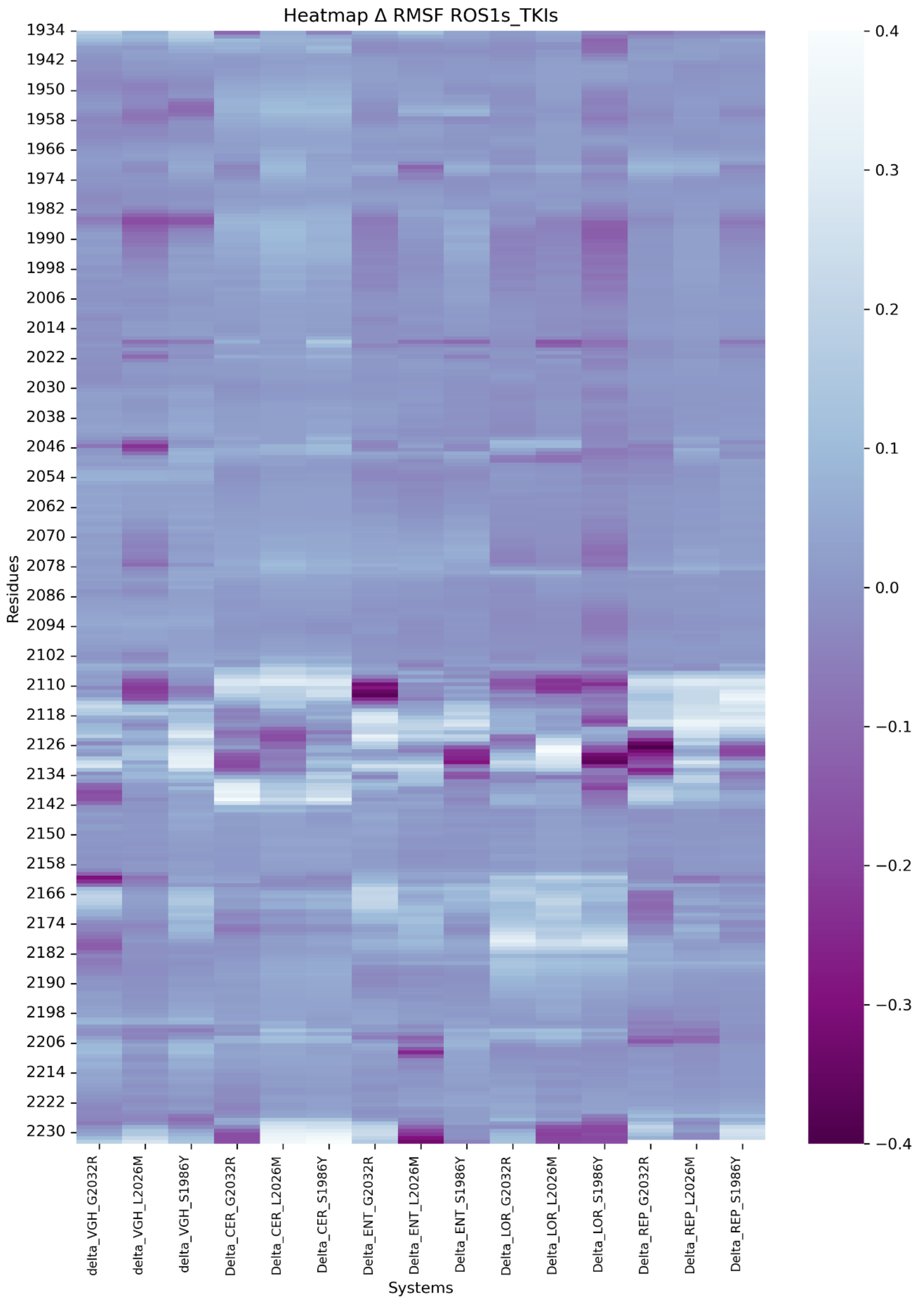

Supplementary Figure 3 ΔRMSF profiles (WT-mutant) highlighted differences in flexibility between mutant models.

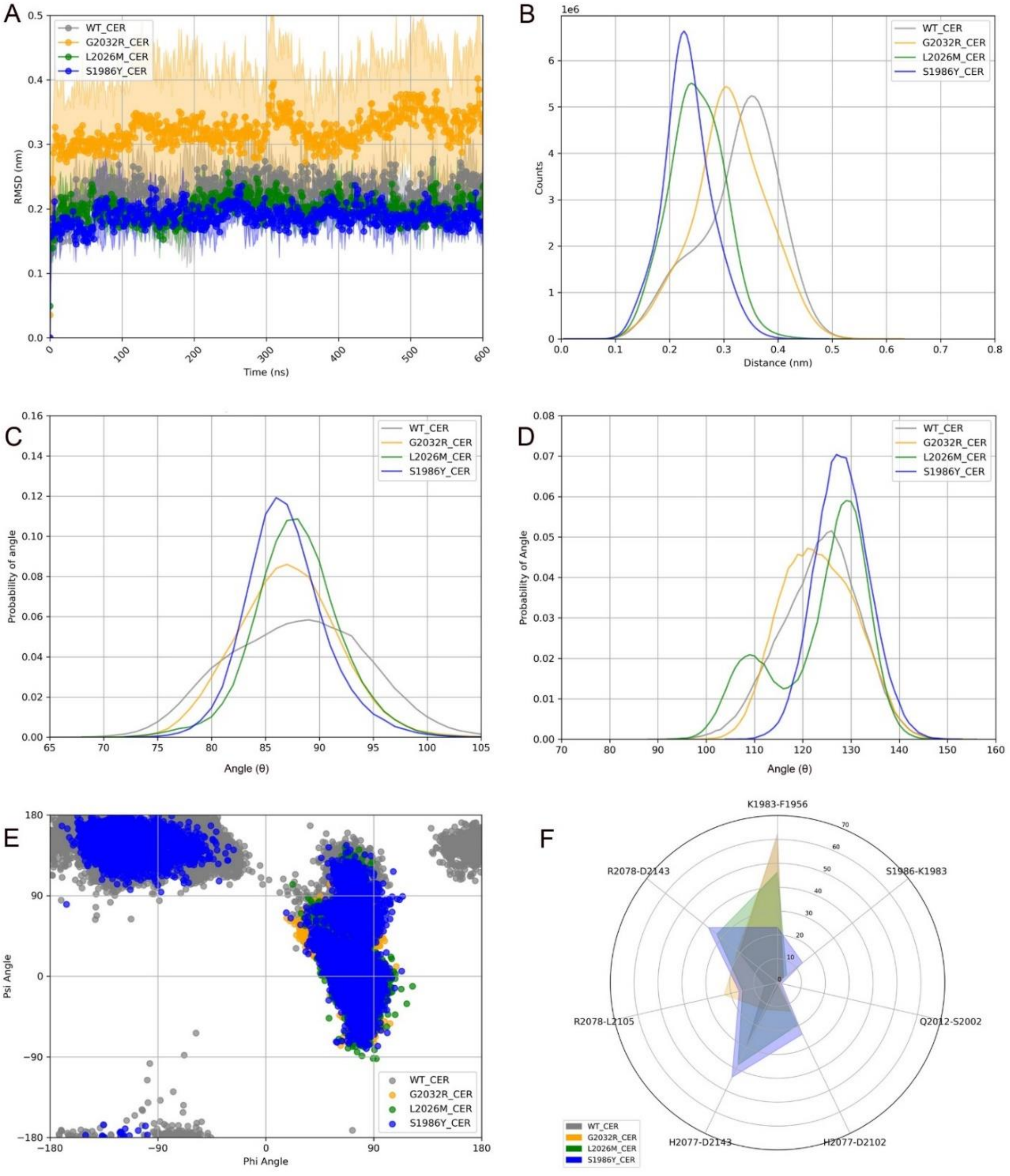

Supplementary Figure 4 Illustrates a comparative analysis of the $ROS1^{WT}$ *and* $ROS1^{mutant}$ *kinase domain using ceretinib-bound simulations.* (A) *RMSD comparison for ROS1 kinase domain residues using backbone atoms.* (B) *RMSD comparison for selected ligands (heavy atoms) and protein (backbone atoms).* (C) *Comparison of the dynamics of three-point angle calculations using residues (1989-2004-2145).* (D) *Comparison of dihedral angle using residues (1982-1954-2003-2112).* (E) *Ramachandran plot comparisons for residue R2078 from the HRD motif.* (F) *Comparison of Hydrogen-bond profiles using selected interactions for* $ROS1^{WT}$ *and* $ROS1^{mutants}$.

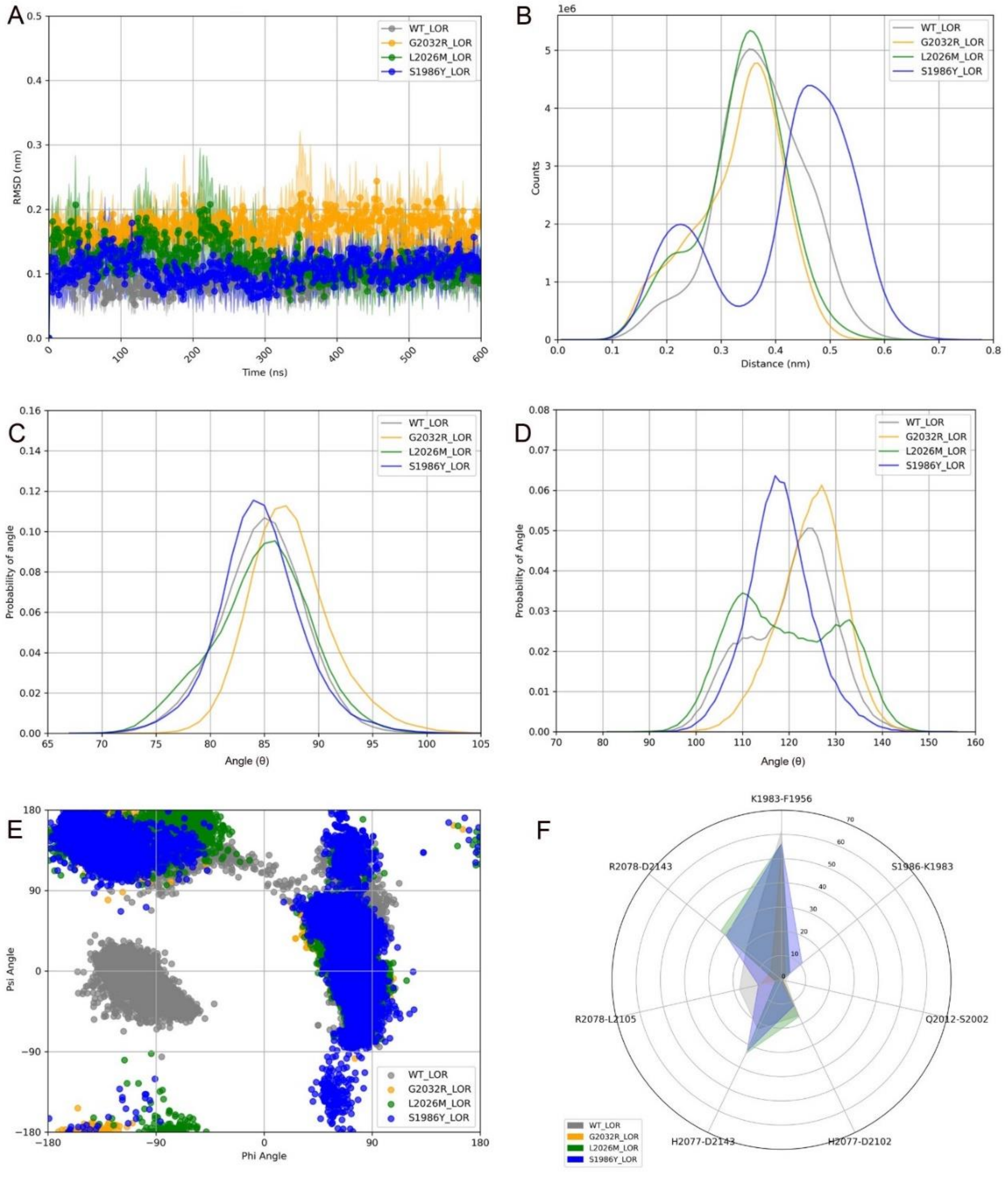

Supplementary Figure 5 Illustrates a comparative analysis of the $ROS1^{WT}$ and $ROS1^{mutant}$ kinase domain using lorlatinib-bound simulations. (A) *RMSD comparison for ROS1 kinase domain residues using backbone atoms.* (B) *RMSD comparison for selected ligands (heavy atoms) and protein (backbone atoms).* (C) *Comparison of the dynamics of three-point angle calculations using residues (1989-2004-2145).* (D) *Comparison of dihedral angle using residues (1982-1954-2003-2112).* (E) *Ramachandran plot comparisons for residue R2078 from the HRD motif.* (F) *Comparison of Hydrogen-bond profiles using selected interactions for $ROS1^{WT}$ and $ROS1^{mutants}$.*

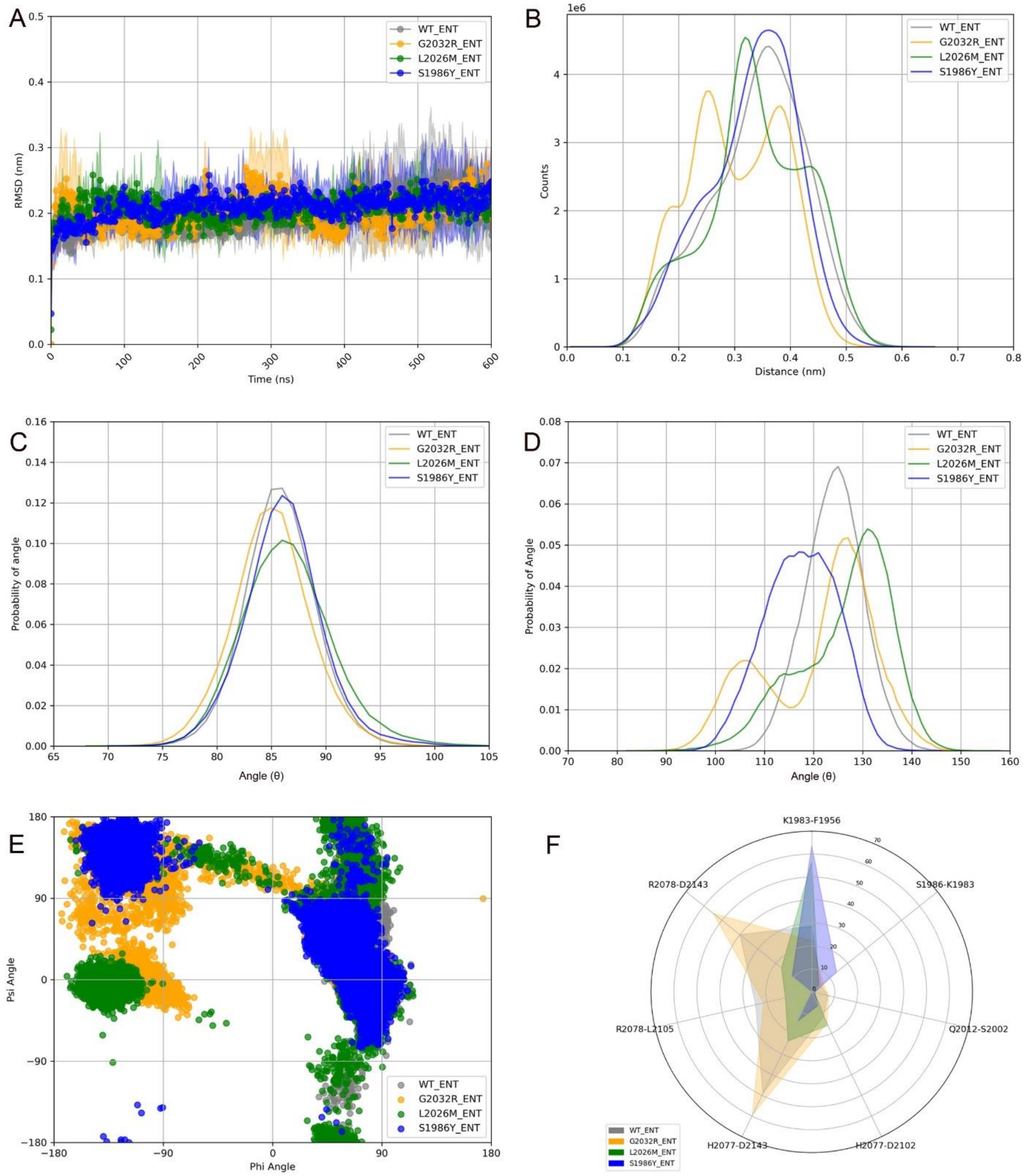

Supplementary Figure 6 Illustrates a comparative analysis of the ROS1^{WT} *and ROS1*^{mutant} *kinase domain using entrectinib-bound simulations.* (A) *RMSD comparison for ROS1 kinase domain residues using backbone atoms.* (B) *RMSD comparison for selected ligands (heavy atoms) and protein (backbone atoms).* (C) *Comparison of the dynamics of three-point angle calculations using residues (1989-2004-2145).* (D) *Comparison of dihedral angle using residues (1982-1954-2003-*

2112). (E) *Ramachandran plot comparisons for residue R2078 from the HRD motif.* (F) *Comparison of Hydrogen-bond profiles using selected interactions for ROS1^{WT} and ROS1^{mutants}.*

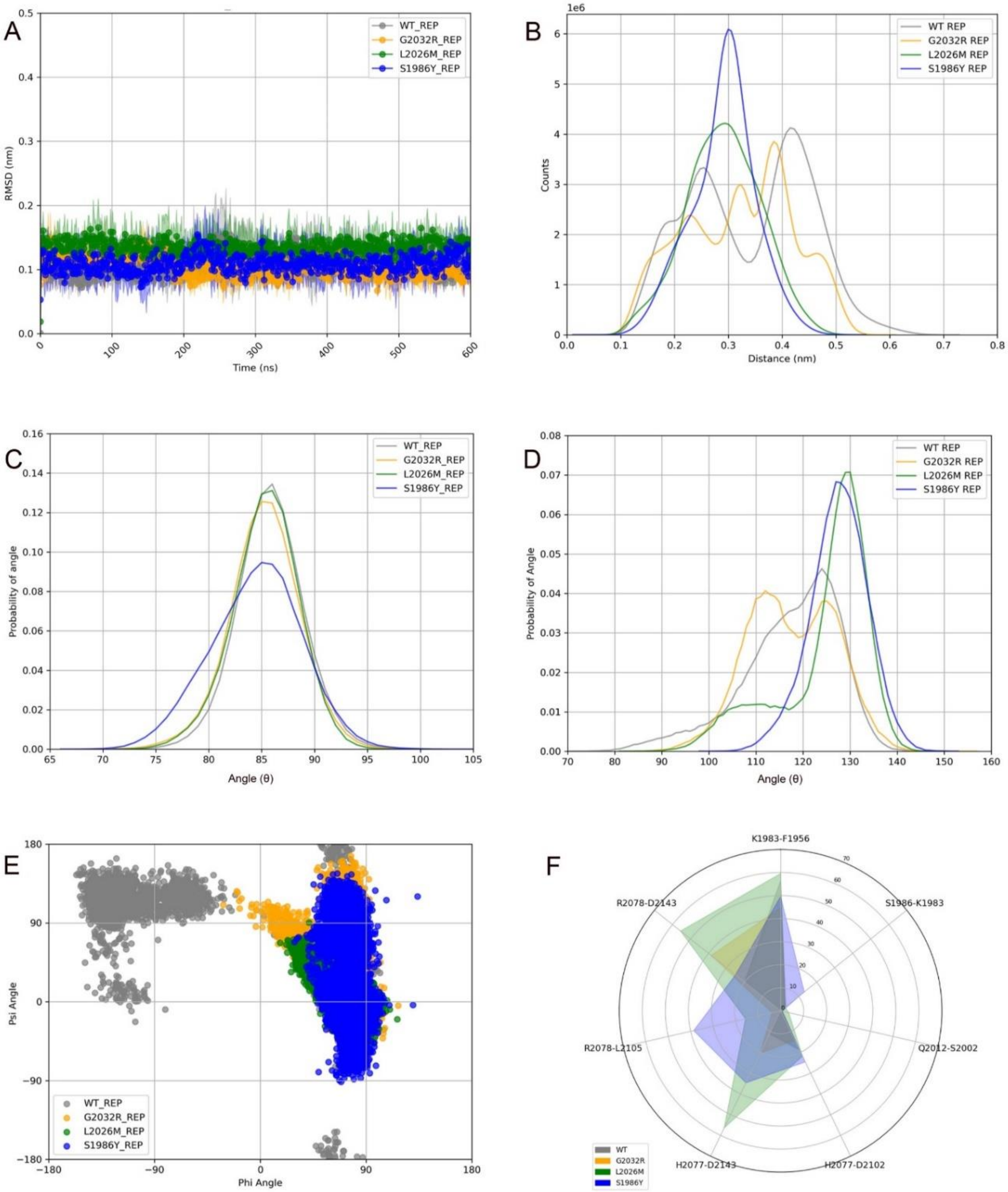

Supplementary Figure 7 Illustrates a comparative analysis of the ROS1^{WT} and ROS1^{mutant} kinase domain using repotrectinib-bound simulations. (A) *RMSD comparison for ROS1 kinase domain residues using backbone atoms.* (B) *RMSD comparison for selected ligands (heavy atoms) and protein (backbone atoms).* (C) *Comparison of the dynamics of three-point angle calculations using residues (1989-2004-2145).* (D) *Comparison of dihedral angle using residues (1982-1954-2003-2112).* (E) *Ramachandran plot comparisons for residue R2078 from the HRD motif.* (F) *Comparison of Hydrogen-bond profiles using selected interactions for ROS1^{WT} and ROS1^{mutants}.*

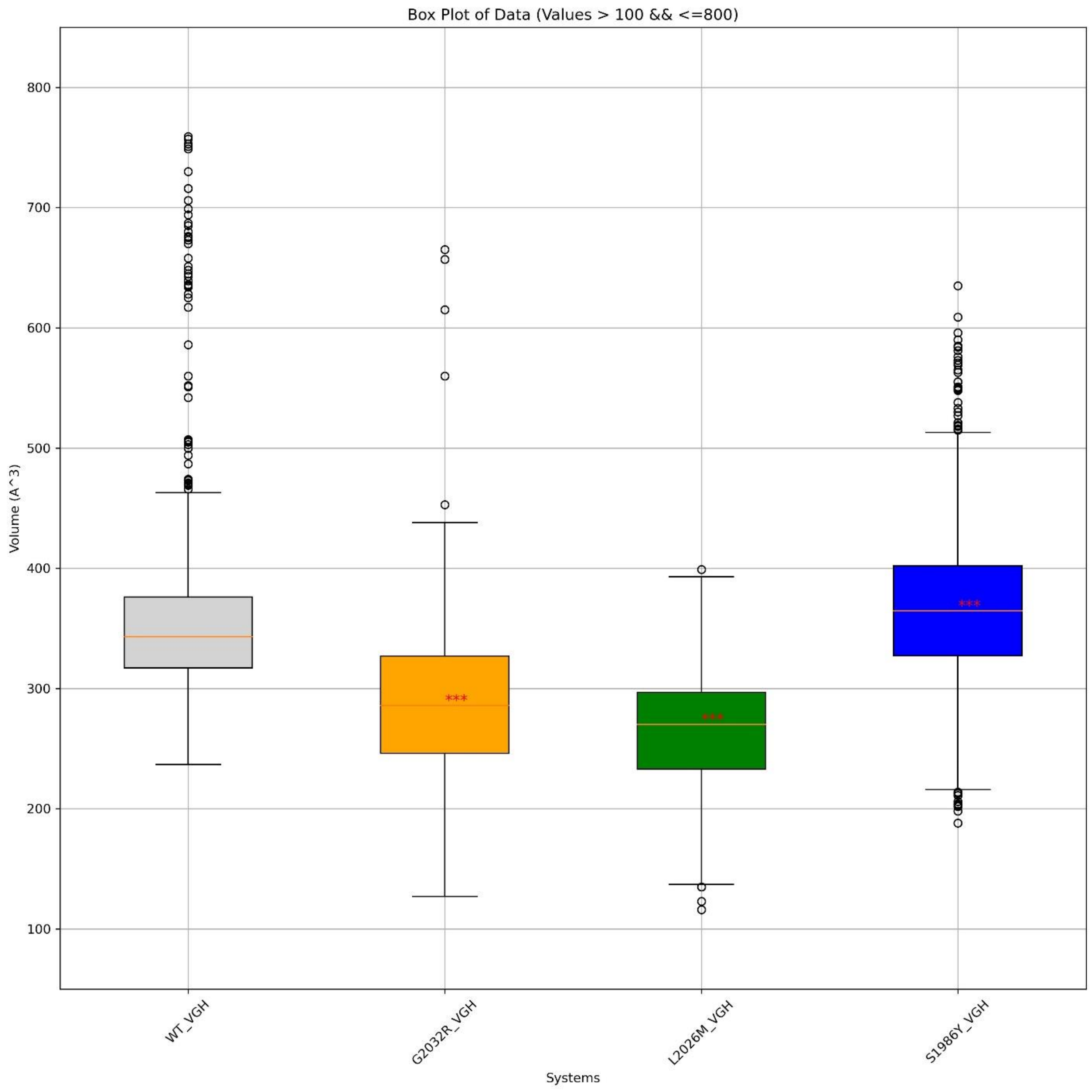

*Supplementary Figure 8 POVME analysis was conducted to calculate the active site pocket for ROS1^{WT} and ROS1^{mutants} using crizotinib-bound simulations. The ROS1^{WT} system is depicted in a grey boxplot, while the ROS1^{mutants} G2032R, L2026M, and S1986Y are shown in orange, green, and blue, respectively. T-test significance values between ROS1^{WT} and ROS1^{mutants} are indicated by ***, **, and * symbols. (*p < 0.05, **p < 0.01, ***p < 0.001, ****p < 0.0001; ns, not significant). In the boxplots, the central line represents the median, boxes indicate the interquartile range (IQR), whiskers extend to 1.5×IQR, and individual points represent outliers.*

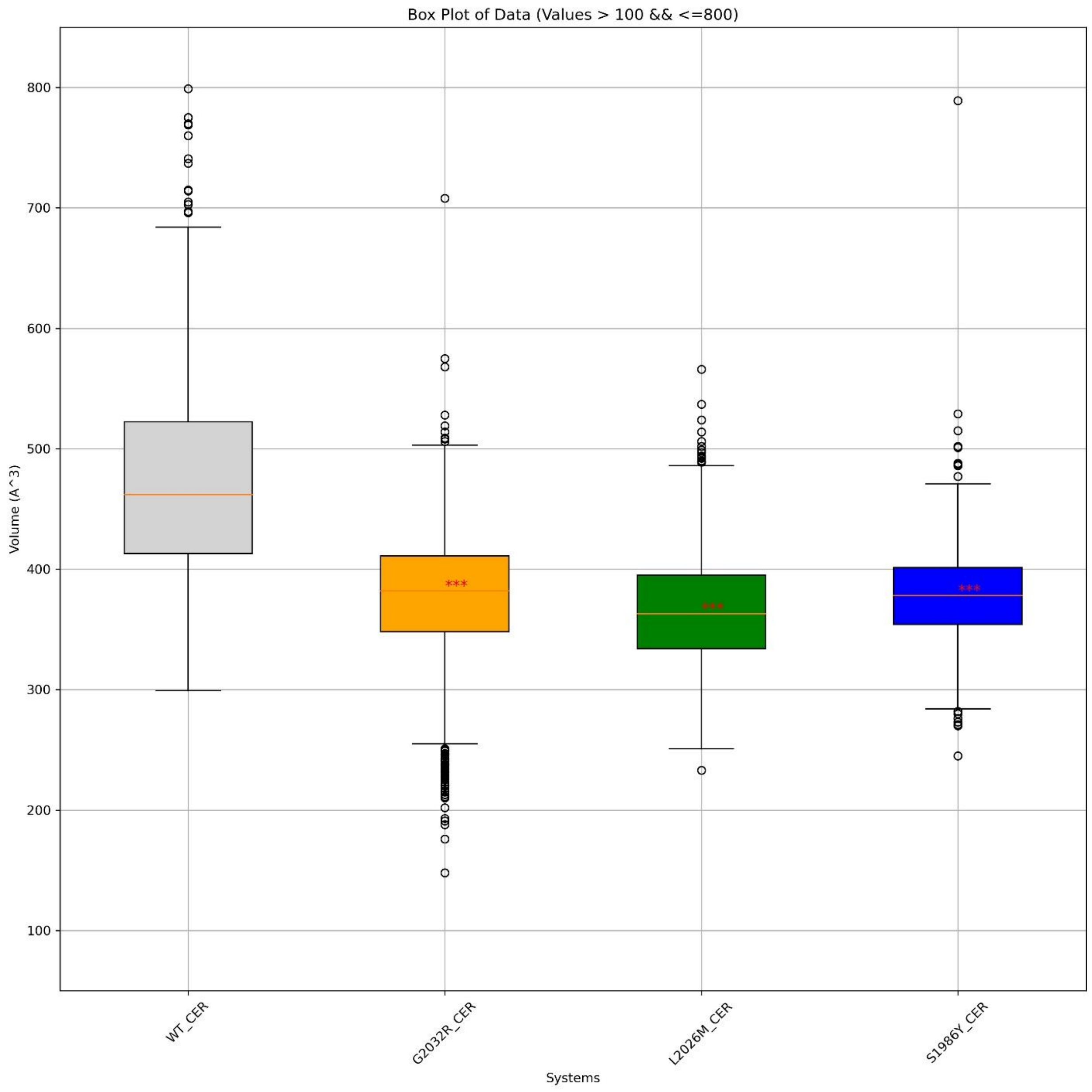

*Supplementary Figure 9 POVME analysis was conducted to calculate the active site pocket for ROS1^{WT} and ROS1^{mutants} using ceretinib-bound simulations. The ROS1^{WT} system is depicted in a grey boxplot, while the ROS1^{mutants} G2032R, L2026M, and S1986Y are shown in orange, green, and blue, respectively. T-test significance values between ROS1^{WT} and ROS1^{mutants} are indicated by ***, **, and * symbols. (*p < 0.05, **p < 0.01, ***p < 0.001, ****p < 0.0001; ns, not significant).*

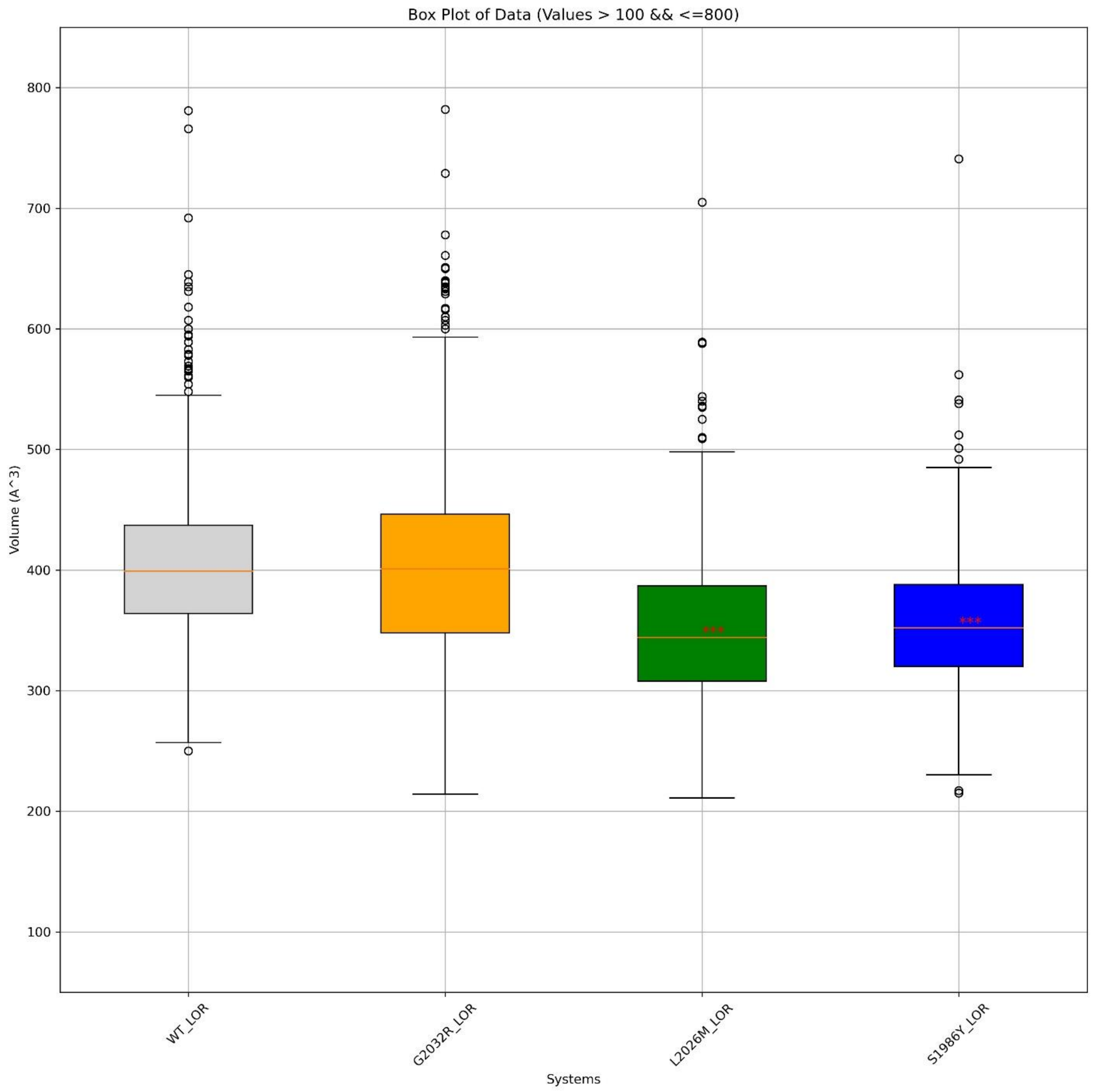

*Supplementary Figure 10 POVME analysis was conducted to calculate the active site pocket for ROS1^{WT} and ROS1^{mutants} using lorlatinib-bound simulations. The ROS1^{WT} system is depicted in a grey boxplot, while the ROS1^{mutants} G2032R, L2026M, and S1986Y are shown in orange, green, and blue, respectively. T-test significance values between ROS1^{WT} and ROS1^{mutants} are indicated by ***, **, and * symbols. (*p < 0.05, **p < 0.01, ***p < 0.001, ****p < 0.0001; ns, not significant).*

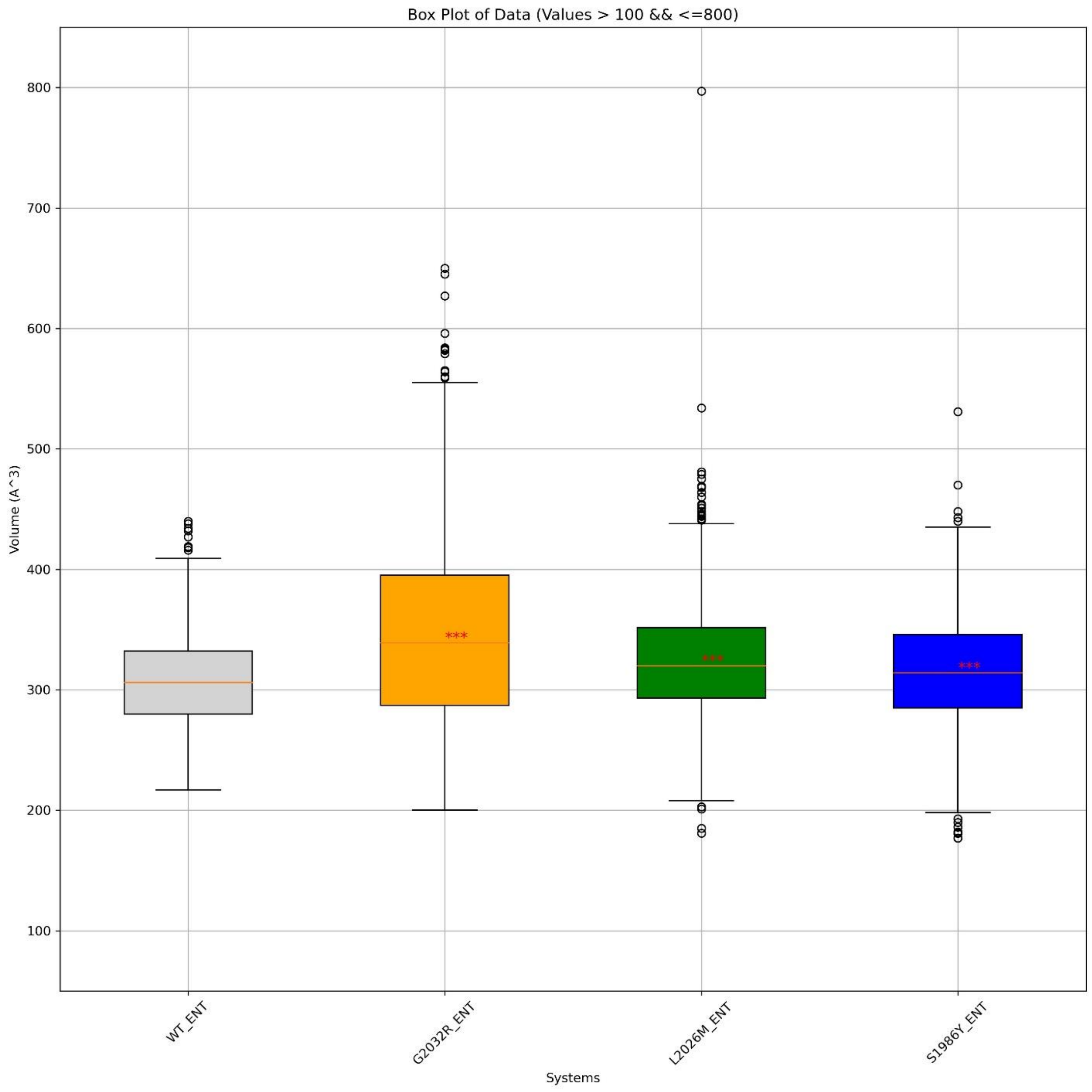

*Supplementary Figure 11 POVME analysis was conducted to calculate the active site pocket for $ROS1^{WT}$ and $ROS1^{mutants}$ using entrectinib-bound simulations. The $ROS1^{WT}$ system is depicted in a grey boxplot, while the $ROS1^{mutants}$ G2032R, L2026M, and S1986Y are shown in orange, green, and blue, respectively. T-test significance values between $ROS1^{WT}$ and $ROS1^{mutants}$ are indicated by ***, **, and * symbols. (*p < 0.05, **p < 0.01, ***p < 0.001, ****p < 0.0001; ns, not significant).*

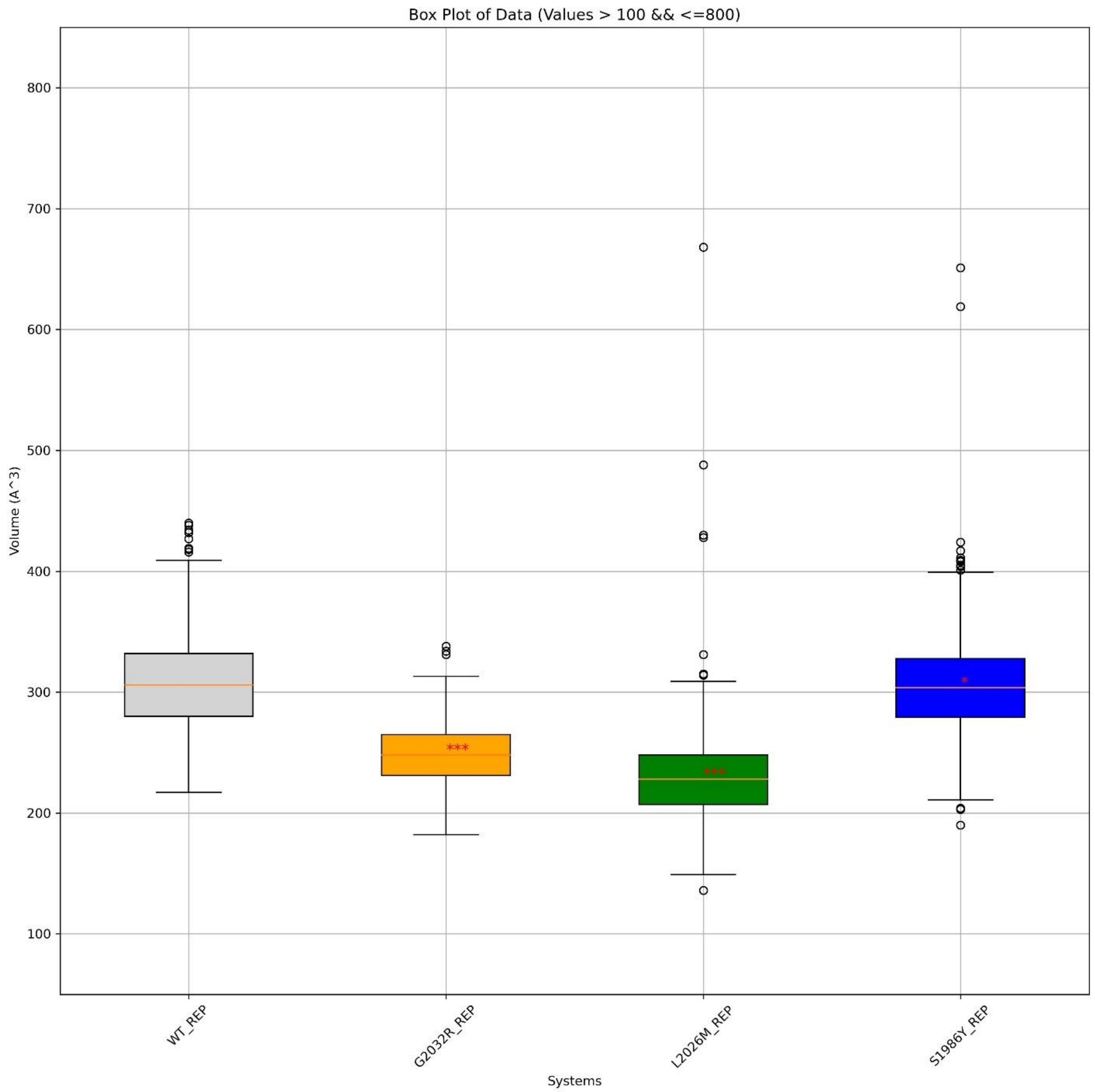

*Supplementary Figure 12 POVME analysis was conducted to calculate the active site pocket for ROS1^{WT} and ROS1^{mutants} using repotrectinib-bound simulations. The ROS1^{WT} system is depicted in a grey boxplot, while the ROS1^{mutants} G2032R, L2026M, and S1986Y are shown in orange, green, and blue, respectively. T-test significance values between ROS1^{WT} and ROS1^{mutants} are indicated by ***, **, and * symbols. (*p < 0.05, **p < 0.01, ***p < 0.001, ****p < 0.0001; ns, not significant).*

A

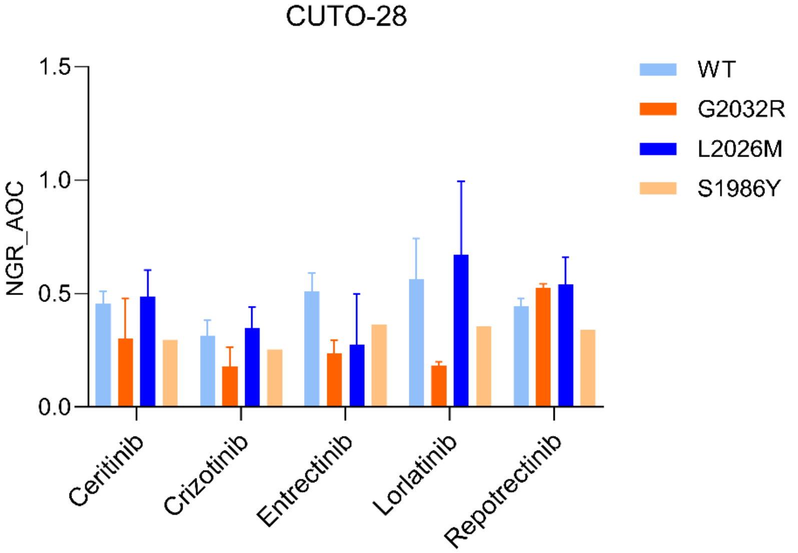

B

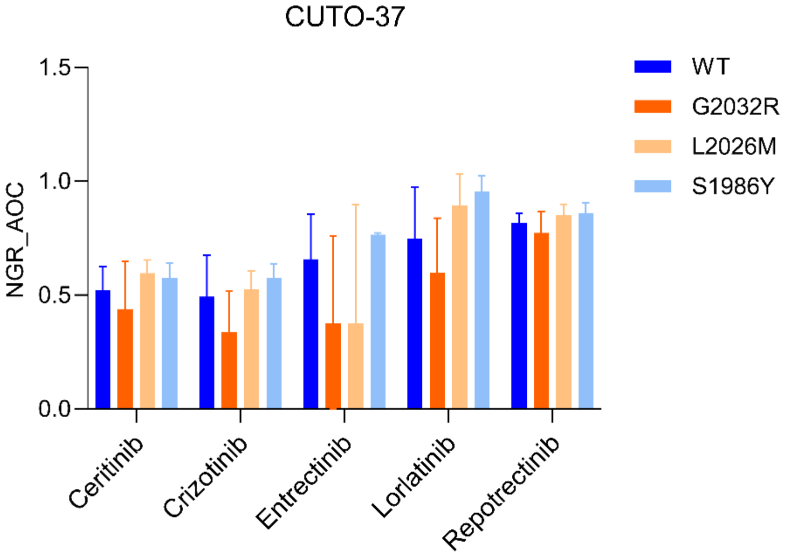

Supplementary Figure 13. Drug response across ROS1 variants in CUTO cell lines. (A–B) Area under the curve (AOC) values derived from normalized growth rate (NGR) dose–response assays in (A) CUTO-28 and (B) CUTO-37 cells treated with Ceritinib, Crizotinib, Entrectinib, Lorlatinib, and Repotrectinib. Bars represent different ROS1 variants (WT, G2032R, L2026M, S1986Y), and error bars (Standard Deviation) indicate variability across replicates. Lower AOC values indicate stronger growth inhibition (greater drug sensitivity), whereas higher AOC values indicate reduced drug efficacy (greater resistance). Differences in AOC reflect mutation-

and cell line–dependent responses to treatment. 2-WAY ANOVA was performed using Dunnett's multiple comparisons test in GraphPad Prism v9. The resulting differences were statistically ns.

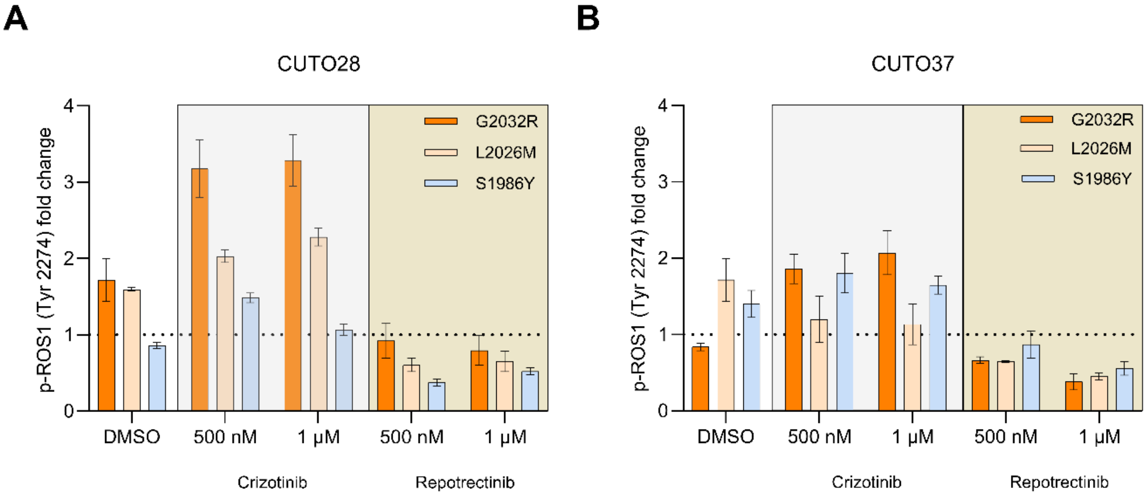

Dunnett's multiple comparisons test

Treatment	Genotype	Summary	P Value
	**G2032R**		
Crizotinib	DMSO vs. 500 nM	****	<0,0001
	DMSO vs. 1 µM	****	<0,0001
Repotrectinib	DMSO vs. 500 nM	**	0,0017
	DMSO vs. 1 µM	***	0,0004
	**L2026M**		
Crizotinib	DMSO vs. 500 nM	ns	0,0902
	DMSO vs. 1 µM	**	0,0058
Repotrectinib	DMSO vs. 500 nM	***	0,0002
	DMSO vs. 1 µM	***	0,0003
	**S1986Y**		
Crizotinib	DMSO vs. 500 nM	*	0,011
	DMSO vs. 1 µM	ns	0,5941
Repotrectinib	DMSO vs. 500 nM	ns	0,0552
	DMSO vs. 1 µM	ns	0,2292

Dunnett's multiple comparisons test

Treatment	Genotype	Summary	P Value
	**G2032R**		
Crizotinib	DMSO vs. 500 nM	***	0,0002
	DMSO vs. 1 µM	****	<0,0001
Repotrectinib	DMSO vs. 500 nM	ns	0,7723
	DMSO vs. 1 µM	ns	0,0866
	**L2026M**		
Crizotinib	DMSO vs. 500 nM	*	0,0467
	DMSO vs. 1 µM	*	0,0239
Repotrectinib	DMSO vs. 500 nM	***	0,0001
	DMSO vs. 1 µM	****	<0,0001
	**S1986Y**		
Crizotinib	DMSO vs. 500 nM	ns	0,1469
	DMSO vs. 1 µM	ns	0,5162
Repotrectinib	DMSO vs. 500 nM	*	0,0411
	DMSO vs. 1 µM	**	0,0015

*Supplementary Figure 14. Effect of ROS1 mutations on p-ROS1 levels following drug treatment. (A–B) Quantification of p-ROS1 (Tyr2274) immunoblot band intensity in (A) CUTO-28 and (B) CUTO-37 cells expressing ROS1 variants (G2032R, L2026M, S1986Y). Cells were treated with DMSO (control), crizotinib (500 nM, 1 µM), or repotrectinib (500 nM, 1 µM). Data are presented as fold change relative to WT after GAPDH normalization. The dashed line indicates baseline (WT = 1). Values above 1 represent increased phosphorylation, whereas values below 1 indicate reduced signaling. Error bars (Standard Deviation) represent variability across replicates. Statistical significance was assessed using two-way ANOVA with Dunnett's multiple comparisons test relative to DMSO for each genotype (*p < 0.05, **p < 0.01, ***p < 0.001, ****p < 0.0001; ns, not significant).*

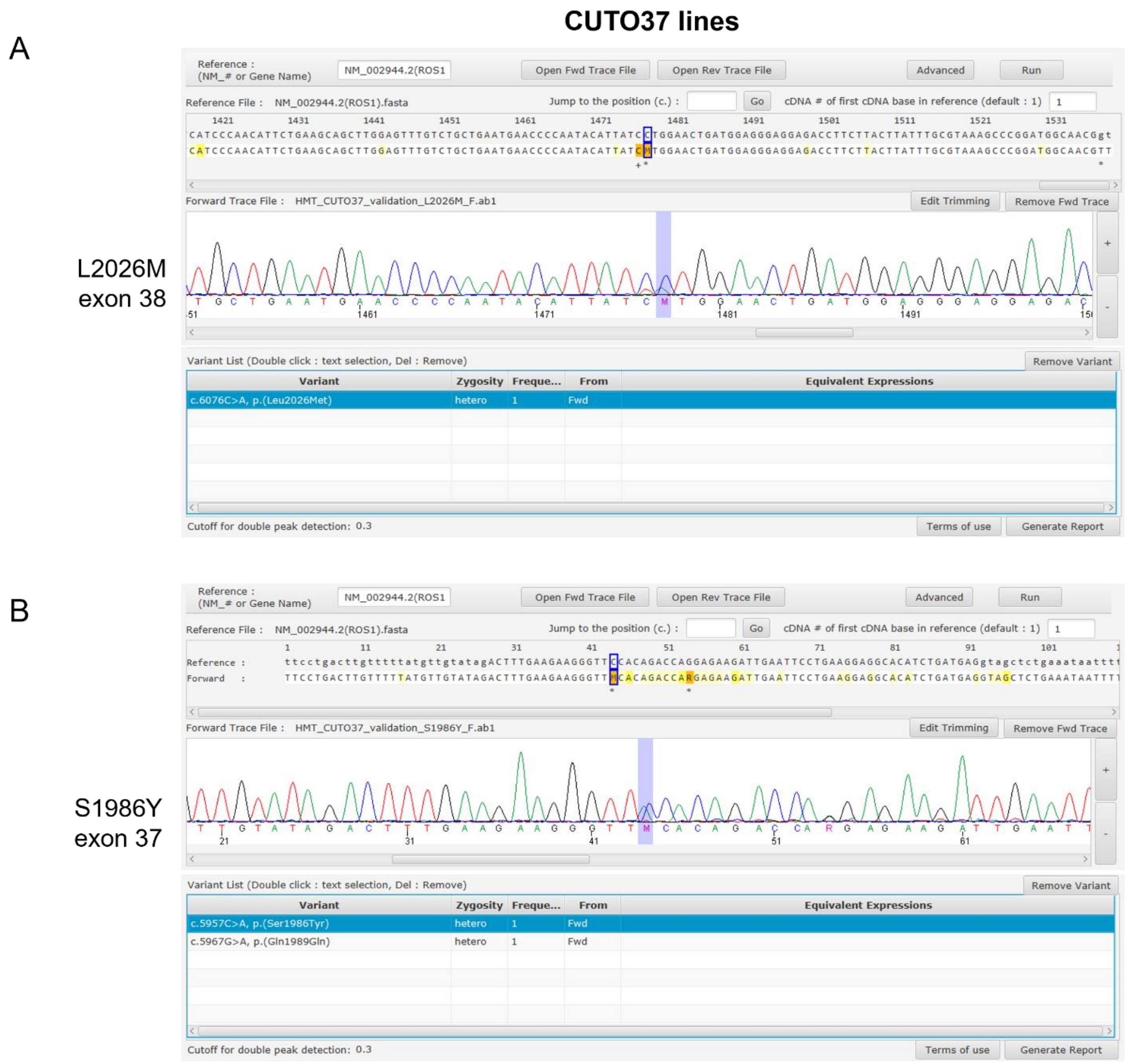

Supplementary Figure 15. Validation of engineered CUTO-37 mutant cell lines. (A) Sequencing validation of the CUTO-37 L2026M cell line showing exclusive editing of CD74–ROS1 rearranged alleles. (B) Sequencing validation of the CUTO-37 S1986Y cell line. An additional silent mutation (c.5967G>A; Gln1989Gln) was introduced in the repair template to disrupt the protospacer adjacent motif (PAM) site and prevent repeated Cas9 cleavage. In both cell lines, sequencing results indicate the presence of at least two ROS1-rearranged alleles, with approximately half carrying the intended mutation.
